# Esculetin inhibits the PI3K/Akt/mTOR pathway and enhances anti-colorectal cancer activity via binding to ENO1

**DOI:** 10.3389/fphar.2025.1627114

**Published:** 2025-07-16

**Authors:** Jianing Ma, Miaomiao Zhang, Shulipan Mulati, Xinhua Nabi, Weiyi Zhang

**Affiliations:** ^1^ School of Pharmacy, Xinjiang Medical University, Urumchi, China; ^2^ Xinjiang Key Laboratory of Natural Medicines Active Components and Drug Release Technology, Urumchi, China; ^3^ Xinjiang Key Laboratory of Biopharmaceuticals and Medical Devices, Urumchi, China; ^4^ Engineering Research Center of Xinjiang and Central Asian Medicine Resources, Ministry of Education, Urumchi, China

**Keywords:** colorectal cancer, esculetin, ENO1, PI3K/Akt/mTOR signaling pathway, DARTS

## Abstract

**Introduction:**

Colorectal cancer (CRC) ranks as the third most prevalent malignant tumor worldwide and is the second leading cause of cancer-related mortality. At present, while its standard treatment consists of a combination of surgery and chemotherapy, metastasis and recurrence are generally associated with a poor prognosis.

**Methods:**

Flow cytometry with Hoechst 33342 staining was employed to detect the changes in cell cycle and apoptosis in CRC cells. The molecular mechanisms of Esc’s antitumor properties were elucidated through network pharmacology, transcriptome sequencing technology, drug affinity responsive target stability (DARTS), and molecular docking. The *in vivo* antitumor effects of Esc were examined using the xenograft mouse model.

**Results:**

In this study, esculetin (Esc) exerted significant anti-proliferative effects across the CRC cell lines HCT116 and HT-29. Furthermore, Esc triggered cell death, arrested the HCT116 cell cycle at the S phase and the HT-29 cell cycle at the G0/G1 phase, inhibited the PI3K/Akt/mTOR signaling pathway, and promoted anti-CRC effects both *in vitro* and *in vivo*. Additional mechanistic investigations revealed that Esc bound to the ENO1 protein and altered its stability. Moreover, silencing ENO1 expression reversed the anti-CRC effect of Esc.

**Discussion:**

This study highlighted the effects of Esc against CRC and clarified that Esc inhibits the PI3K/Akt/mTOR signaling pathway and enhances the anti-CRC activity by binding to ENO1, suggesting that ENO1 may become a potential target for the treatment of CRC. It may strengthen the evidentiary foundation for developing novel antitumor agents with enhanced efficacy and reduced toxicity.

## Graphical Abstract

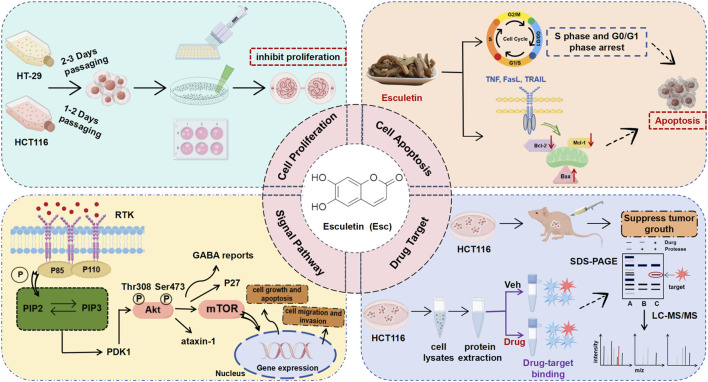

## 1 Introduction

Colorectal cancer (CRC) ranks among the most prevalent malignant tumors globally, with its incidence steadily rising, thereby posing a significant threat to human health. Notably, it is a multifactorial and multistage condition that originates in the epithelial cells of the colon or rectum as a result of genetic mutations (Sninsky et al., 2022). Depending on the site of origin, CRC can be classified as either colon or rectal cancer (closer to the anus) (Mahmoud, 2022). Due to their shared clinical characteristics, colon and rectal cancers are frequently grouped. The standard treatment protocol for CRC primarily involves surgical resection, potentially combined with adjuvant radiotherapy or chemotherapy. In recent years, CRC mortality rates have shown a gradual decline, attributable to improvements in early detection methods and therapeutic advancements, including chemotherapy, targeted therapy, and immunotherapy (Aykut and Lidsky, 2023). Among them, XELOX (oxaliplatin combined with capecitabine), FOLFOX (combination of 5-fluorouracil (5-Fu), calcium folinate, and oxaliplatin), and FOLFIRI (combination of 5-Fu, calcium folinate, and irinotecan) have been extensively used as first-line chemotherapy regimens for advanced CRC. Furthermore, cetuximab and bevacizumab have demonstrated favorable efficacy and are primarily used in combination with systemic chemotherapy (Kvakova et al., 2022). Although chemotherapeutic agents demonstrate high efficacy in treating malignant tumors, their ability to induce cardiovascular complications presents a significant clinical concern (Quryshi et al., 2018). In 70% of CRC cases with advanced-stage disease, patients frequently develop myocardial mitochondrial dysfunction secondary to systemic inflammatory responses. This pathological process ultimately compromises the cardiac energy metabolism (Smuder et al., 2020; Lee et al., 2020). Notably, commonly used CRC therapeutic agents, including 5-Fu, capecitabine, and targeted therapies like cetuximab, exhibit intrinsic cardiotoxic properties. Chronic administration of these drugs may result in structural myocardial damage, progressive cardiac dysfunction, and subsequent cardiovascular events, all of which contribute to diminished overall survival rates in patient populations (Rosa et al., 2016). Therefore, it is imperative to develop new candidate drugs.

Esculetin (Esc), a coumarin derivative, is a compound primarily extracted from *Artemisia capillaris*, *Citrus limonia*, and *Euphorbia lathyris*. Previous studies have identified its multifaceted biological properties, including suppression of free radical generation, reduction of inflammatory marker levels, modulation of glycemic responses, and regulation of gene expression associated with cancer progression and hepatic dysfunction (Garg et al., 2022). Indeed, it exerts a wide range of pharmacological effects, including the dual regulation of cell death, anti-diabetic effects (Kadakol et al., 2015), and anti-inflammatory effects (Ozal et al., 2018), which are partially linked to their antioxidant nature (Pruccoli et al., 2020). Additionally, it has been noted to suppress the proliferation of various tumor cells, including human pancreatic cancer cells (Asmat et al., 2016), leukemia cells (Rubio et al., 2017), gastric cancer cells (Wang et al., 2017), laryngeal cancer cells (Zhang et al., 2019), and oral squamous cancer cells (Cho et al., 2015). Although previous studies have reported that Esc can suppress CRC cell proliferation through reactive oxygen species-induced mitochondrial apoptosis (Kim et al., 2015) or targeting the Axin2/E-cadherin axis (Kim et al., 2018), the specific targets and mechanisms remain underexplored and elusive.

Enolase 1 (ENO1) is an essential enzyme in glycolysis implicated in various physiological processes. According to an earlier study, the ENO1 signaling pathway could be a viable target for cancer therapies (Park et al., 2016). For instance, estrogen enhances cell motility by triggering stromal cells in prostate cancer to release ENO1 through the paracrine signaling axis (Yu et al., 2012). In breast cancer cells, ENO1 and associated proteins can reduce the levels of heat shock proteins (Balasubramani et al., 2011). Additionally, ENO1 expression is upregulated by glucose transporters and glycolytic enzymes, thereby promoting the Warburg effect (Cairns et al., 2011). Moreover, ENO1 expression has been described to influence the cell cycle (Zhang et al., 2020).

This study demonstrated that Esc triggered cell cycle arrest and CRC apoptosis via multiple signaling pathways and exerted anti-CRC effects both *in vitro* and *in vivo*. Additionally, the drug affinity responsive target stability (DARTS) assay identified ENO1 as a target of Esc in CRC. The binding of Esc with ENO1 altered its stability. Lastly, silencing ENO1 expression reversed the anti-CRC effect of Esc through the PI3K/Akt/mTOR signaling pathway. Taken together, these findings could offer a novel approach for the management of CRC.

## 2 Materials and methods

### 2.1 Reagents and antibodies

The Esc (HPLC≥98%, B20991) was purchased from Yuanye (Shanghai, China). Antibodies against Bax (bs-0127R), Bcl-2 (bs-0032R), and Mcl-1 (bs-23315R) were procured from Bioss (Beijing, China). Antibodies against AKT (bsm-33278M), P-AKT (bs-2720R), MTOR (bs-1992R), P-MTOR (bs-3495R), PI3K (bs-6417R), P-PI3K (bs-6417R), STAT3 (bsm-33218M) and P-STAT3 (bs-1658R), anti-GADPH (bsm-52262R), ENO1 (bs-3978R), and β-actin (bs-0061R) were sourced from Bioss (Beijing, China). The Protein Rapid Silver Staining Kit (R21273) was purchased from Yuanye (Shanghai, China). Pronase E (P8360) was acquired from Solarbio (Beijing, China). Recombinant lentiviral vectors (1348084) were obtained from Genechem (Shanghai, China).

### 2.2 Cell culture and cell viability assessment

HCT116 cells (CVCL-0291) were cultured in DMEM, while HT-29 cells (CVCL-0320) were cultured in McCoy’s 5A medium. All media were supplemented with 10% fetal bovine serum, 100 units/mL penicillin, and streptomycin and cultured at 37°C in an atmosphere containing 5% CO_2_. The Cell Counting Kit-8 assay was performed to assess cell viability. All human CRC cell lines were authenticated by short tandem repeat (STR) profiling. All experiments were conducted using mycoplasma-free cells. Esc (molecular weight 178.14) was dissolved in DMSO to a final concentration of 10 mM stock solution. The stock solution was subsequently diluted to desired concentrations in 1× PBS and used for experiments.

### 2.3 Colony formation assay

HCT116 and HT-29 cells were seeded in six-well culture plates (1000 cells/well). After 48 h, Esc (0–20 μM) was introduced into the medium, and the resulting mixture was incubated for an additional 12 days. Afterward, HCT116 cells were washed with PBS, fixed in 4% methanol for 15 min, and stained with Giemsa for 20 min, following which colonies were imaged.

### 2.4 Cell migration assay

HCT116 and HT-29 cells were seeded at a density of 5 × 10^5^ cells per well into six-well plates. Upon reaching 90% confluence, a scratch with a uniform width was made using a sterile pipette tip, following which the cells were washed with PBS. Then, wound mages were captured, and the migratory capacity of cells was examined after 48 h. The cell migration rate was calculated as follows: cell migration rate (%) = [1 − (0 h Scratch width/48 h Scratch width)].

### 2.5 Cell cycle assay

HCT116 and HT-29 cells were treated with Esc (0–200 µM) for 24 h. Next, the cells were collected, washed in PBS, and centrifuged. Cell pellets were subsequently fixed overnight with pre-cooled 75% (v/v) ethanol at −20°C. The proportions of cells across the G0/G1, G2/M, and S phases were quantitatively analyzed using a cell cycle kit and flow cytometry.

### 2.6 Hoechst 33342 staining assay

HCT116 and HT-29 cells were incubated with 0–200 µM Esc for 24 h, following which they were washed in PBS and fixed with 4% methanol. Then, they were stained with Hoechst 33342. Lastly, apoptotic cells were visualized under an inverted fluorescence microscope.

### 2.7 Western blotting assay

HCT116 and HT-29 cells were treated with Esc (0–200 µM) for 24 h. Afterward, whole-cell extracts were prepared, and proteins were separated by SDS-PAGE, transferred to polyvinylidene difluoride (PVDF) membranes, and incubated with protein-specific antibodies, followed by incubation with HRP-conjugated secondary antibodies. The Ctrl group indicates the addition of 0.1% DMSO solution, while the 0 Esc (μM) group represents samples that received no treatment.

### 2.8 Network pharmacology analysis

PubChem software was used to derive the three-dimensional (3D) structure of Esc, while the Swiss Target Prediction and PharmMapper databases were utilized to predict putative target proteins. The identified 45 target proteins were then imported into the STRING and Metascape databases to construct PPI networks. Finally, KEGG and GO enrichment analyses were performed.

### 2.9 Transcriptomic analysis

The cDNA libraries were sequenced on the Illumina sequencing platform by Metware Biotechnology Co., Ltd. (Wuhan, China). The experimental procedures were as follows. Cell samples (with the control group treated with DMSO and the CA group treated with 50 µM Esc for 24 h) were subjected to RNA extraction using the TRIzol method. RNA integrity was rigorously assessed using the Qsep400 Bioanalyzer. Subsequent cDNA synthesis and library preparation were performed by Metware Biotechnology Co., Ltd. (Wuhan, China). Following successful quality control of the libraries, high-throughput sequencing was conducted on the Illumina platform.

### 2.10 DARTS assay

HCT116 cells were lysed in a lysate containing fresh protease inhibitors for 20 min, and protein concentration was quantified using a bicinchoninic acid assay (BCA) kit. Then, the supernatant was assigned to three groups, namely the Ctrl and drug delivery groups. Thereafter, different concentrations of Esc were added to the lysates of the drug delivery groups, and the mixtures were incubated at 37°C for 2 h. In the Ctrl group, an equivalent volume of 1×TNC buffer was added. Subsequently, Pronase E was added, and the mixture was incubated for 1 h at 37°C. After incubation, 5× loading buffer was added, and the mixture was boiled for 5 min. The samples were subjected to SDS-PAGE electrophoresis and silver staining.

### 2.11 Cellular thermal shift assay (CETSA)

HCT116 cells were treated with Esc (50 μM) for 24 h. Then, they were exposed to different temperatures using a metal bath. ENO1 protein expression was detected via Western blot analysis.

### 2.12 Molecular docking and molecular dynamics

The 3D structure of esculetin was retrieved from the PubChem database and subsequently subjected to energy minimization using the MMFF94 force field. The crystal structure of ENO1 (PDB ID: 2PSN) was obtained from the Protein Data Bank (PDB). Molecular docking simulations were carried out using AutoDock Vina 1.2.3 software. Molecular dynamics simulations were performed with the AMBER 22 software package, utilizing the docked small molecule-protein complex as the initial structure.

### 2.13 Xenografts *in vivo*

In this study, 6–8-week-old male BALB/c-nude mice (16–20 g, specific pathogen-free grade, SPF) were obtained from the Beijing Viton Lihua Laboratory Animal Technology Co. (SYXK 2023-0004, Beijing, China), and each nude mouse was subcutaneously injected with 10 million HCT116 cells. After 5 days, the mice were divided into five groups. Briefly, nude mice were treated with 5-Fu (10 mg/kg/^2^ days) and Esc (20 mg/kg/day, 40 mg/kg/day, and 80 mg/kg/day) via daily oral gavage for 13 days, whereas the negative control group was administered saline. Tumor volume was recorded every 3 days and calculated as length × width^2^/2.

### 2.14 Constructing low ENO1-expressing HCT116 cells

Recombinant lentiviral vectors were purchased from Genechem (Shanghai, China). Transfection procedures were carried out using HitransG P (a virus infection enhancer, MOI = 50), and subsequent experimental analyses were initiated 72 h post-transfection. To ensure a stable selection of transfected cells, cultures were maintained in complete medium supplemented with 2 μg/mL puromycin (01,100,205, Labgic Technology, Beijing, China).

### 2.15 Statistical analysis

Data were expressed as mean values ±SD. Differences between the two groups were assessed using t-tests, and multi-group comparisons were analyzed using analysis of variance (ANOVA). *p* < 0.05 was considered statistically significant.

## 3 Results

### 3.1 Esc inhibited the proliferative abilities of HCT116 cells and HT-29 cells *in vitro*

**FIGURE 1:**
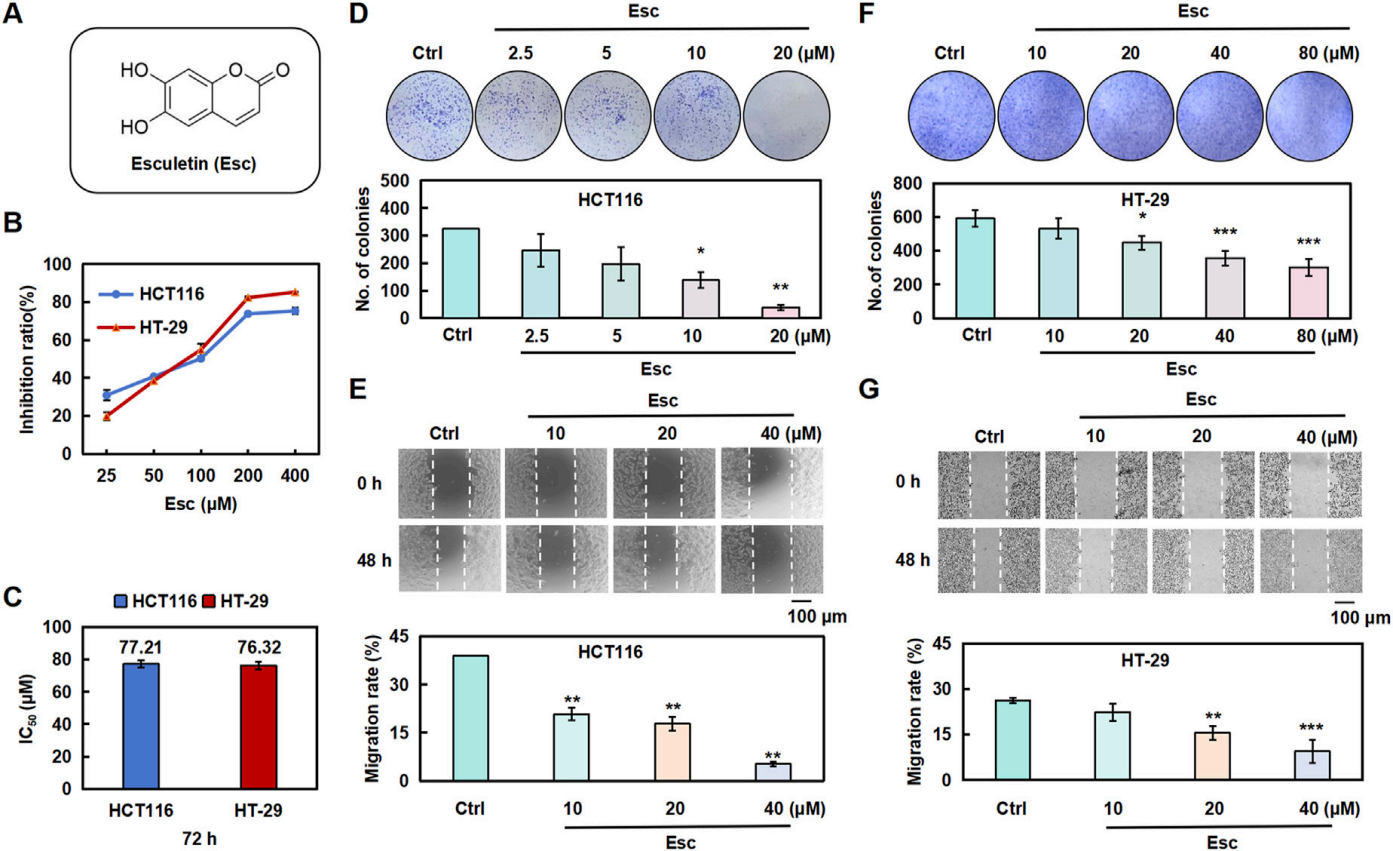
Esc inhibited the proliferative abilities of HCT116 and HT-29 cells *in vitro*. **(A)** Structure of Esc. **(B,C)** HCT116 and HT-29 cells were exposed to Esc (0–400 μM) for 72 h. Cell viability was assessed using the CCK-8 assay, and the IC_50_ values of Esc were calculated. **(D-F)** HCT116 and HT-29 cells were exposed to 0–80 μM Esc for 12 days, followed by Giemsa staining; colony counts are displayed in the histogram. **(E–G)** HCT116 and HT-29 cells were exposed to 0–40 μM Esc for 24 h, following which cell migration rates were analyzed, as depicted in the histogram (n = 3). Data are expressed as means ± SD. *p < 0.05, **p < 0.01 versus the Ctrl group.

To evaluate the impact of Esc on cell proliferation, HCT116 cells and HT-29 cells were exposed to Esc for 24 h, 48 h, and 72 h. The findings indicated that Esc markedly suppressed the proliferative abilities of HCT116 and HT-29 cells in a dose- and time-dependent manner (Figure 1B). The IC_50_ values of Esc against HCT116 cells and HT-29 cells were 77.21 μM and 76.3 μM after 72 h (Figure 1C). As illustrated in Figures 1D–G, the results of the colony formation and migration assays revealed that the colony-forming and migratory abilities of HCT116 cells and HT-29 cells were significantly lower in groups treated with Esc than in the Ctrl group. These results collectively suggest that Esc inhibited the growth of HCT116 cells and HT-29 cells *in vitro*.

### 3.2 Esc induced cell cycle arrest and apoptosis in HCT116 and HT-29 cells

**FIGURE 2:**
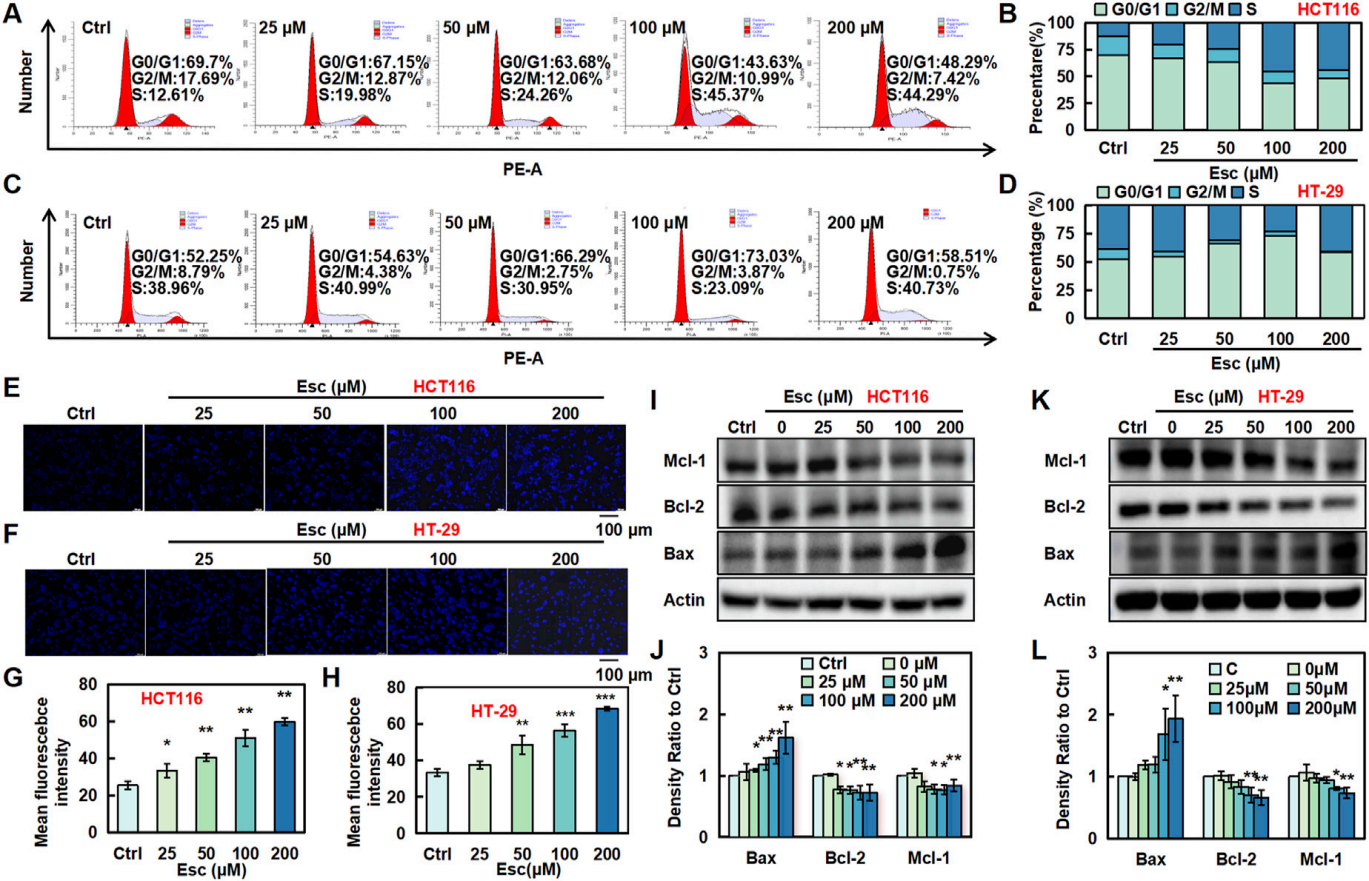
Esc induced cell cycle arrest and apoptosis in HCT116 and HT-29 cells. **(A–D)** HCT116 and HT-29 cells were exposed to 0–200 μM Esc for 24 h, and flow cytometry was performed. **(E–H)** HCT116 and HT-29 cells were exposed to 0–200 µM Esc for 24 h, and alterations in nuclear morphology were examined. **(I–L)** HCT116 and HT-29 cells were exposed to 0–200 µM Esc for 24 h, and protein expression levels were measured. Histograms present density ratios (n = 3). Data are expressed as means ± SD. *p < 0.05, **p < 0.01 versus the Ctrl group.

To investigate the effects of Esc on the cell cycle, flow cytometry was conducted to examine the cell cycle distribution. As illustrated in Figures 2A–D, the proportion of HCT116 cells in the S phase rose from 12.61% to 44.29% with increasing Esc concentrations, and the proportion of HT-29 cells in the G0/G1 phase similarly increased. To explore the relationship between the cytotoxic effects of Esc and apoptosis, Hoechst 33342 staining and Western blotting assays were carried out. As depicted in Figures 2E–H, Esc enhanced blue fluorescence in the nuclear chromatin of HCT116 and HT-29 cells. Additionally, Esc significantly decreased the levels of the anti-apoptotic proteins Mcl-1 and Bcl-2 while significantly increasing the levels of the pro-apoptotic protein Bax (Figures 2I–L). These findings conjointly implied that Esc induced cytotoxicity by promoting cell cycle arrest and apoptosis.

### 3.3 Esc exerted anti-CRC effects by inhibiting the PI3K/AKT/Stat3/mTOR pathways

**FIGURE 3:**
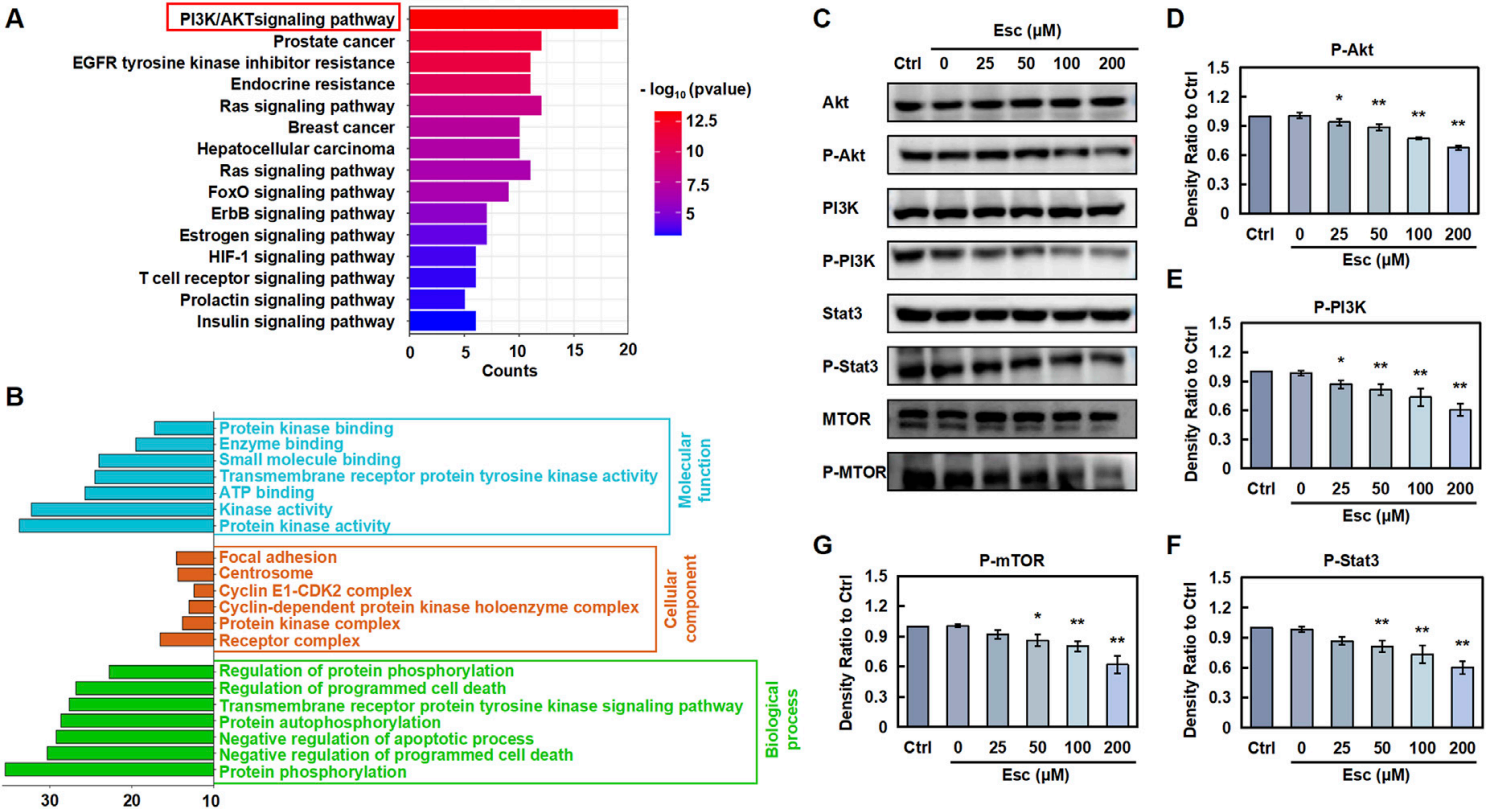
Esc inhibits the PI3K/AKT/Stat3/mTOR pathways to exert anti-CRC effects **(A)** KEGG analysis. **(B)** GO analysis. **(C)** HCT116 cells were exposed to 0–200 µM Esc for 24 h, and the expression levels of the indicated proteins were determined. **(D–G)** Histograms illustrate density ratios (n = 3). Data are expressed as means ± SD. *p < 0.05, **p < 0.01 versus the Ctrl group.

To validate the mechanism by which Esc exerts anti-CRC effects, potential pathways were analyzed via KEGG pathway analysis. As presented in Figure 3A, Esc was involved in multiple signaling pathways. Among them, we found that Esc had the highest significance with the PI3K/Akt signaling pathway. These activated pathways, in turn, stimulate cell growth and escape from apoptosis (He et al., 2021). Meanwhile, GO analysis revealed that aberrant activation of these pathways contributed to tumor progression (Figure 3B). Notably, the Western blot results unveiled that Esc inhibited the phosphorylation of Akt, PI3K, mTOR, and Stat3 without altering their overall protein levels (Figures 3C–G). Thus, the aforementioned results indicated that Esc exerts anti-CRC effects by inhibiting the PI3K/Akt/Stat3/mTOR signaling pathway.

### 3.4 Transcriptomics explored Esc’s targets and functional characteristics

**FIGURE 4:**
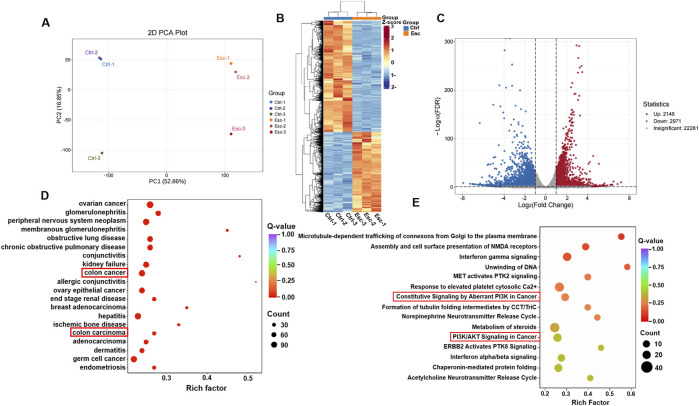
Transcriptomic insights into Esc’s biological targets and functional impact. **(A)** PCA separation of the Ctrl and Esc-treated cells. **(B)** Differential gene expression heatmap; **(C)** Volcano plot showing differential genes; **(D)** DO analysis scatter plot for CRC; **(E)** Reactome pathway scatter plot highlighting PI3K/AKT signaling.

Transcriptomics was employed to analyze the complex interactions between the drug and its targets, providing a comprehensive evaluation. To further validate the molecular mechanisms underlying Esc’s antitumor properties in CRC, we conducted a comprehensive gene expression analysis of HCT116 cell samples treated with Esc using RNA sequencing. HCT116 cells were treated with 50 µM Esc for 24 h, followed by transcriptomic sequencing. The expression violin plot (Supplementary Figure S1A) and sample correlation plot (Supplementary Figure S1B) revealed low dispersion of gene expression within groups, indicating small intra-group variations, confirming the suitability of the Ctrl and Esc groups for inter-group differential gene analysis. The results of principal component analysis (PCA) (Figure 4A) and differential gene cluster heatmaps (Figure 4B) collectively showed distinct separation between the Ctrl and Esc groups. A volcano plot of differential gene expression (Figure 4C) identified a total of 5,117 differential genes between the Esc and Ctrl groups, with 2,146 upregulated and 2,971 downregulated genes.

A scatter plot was used for disease ontology (DO) analysis of differential genes (Figure 4D). The DO enrichment was assessed based on the Rich factor, the Q-value, and the number of differential genes associated with a particular disease. The KEGG enrichment analysis of the differentially expressed genes revealed a more significant enrichment of the PI3K/AKT signaling pathway (Supplementary Figure S1C). The reactome enrichment scatter plot (Figure 4E) highlighted pathways, such as constitutive signaling by aberrant PI3K in cancer and PI3K/AKT signaling in cancer, which are relevant to Esc’s anti-CRC effects. These findings suggest that Esc likely exerts its effects by modulating these signaling pathways and target processes to intervene in the development of CRC.

### 3.5 Esc targeted ENO1 and reduced its protein stability

**FIGURE 5:**
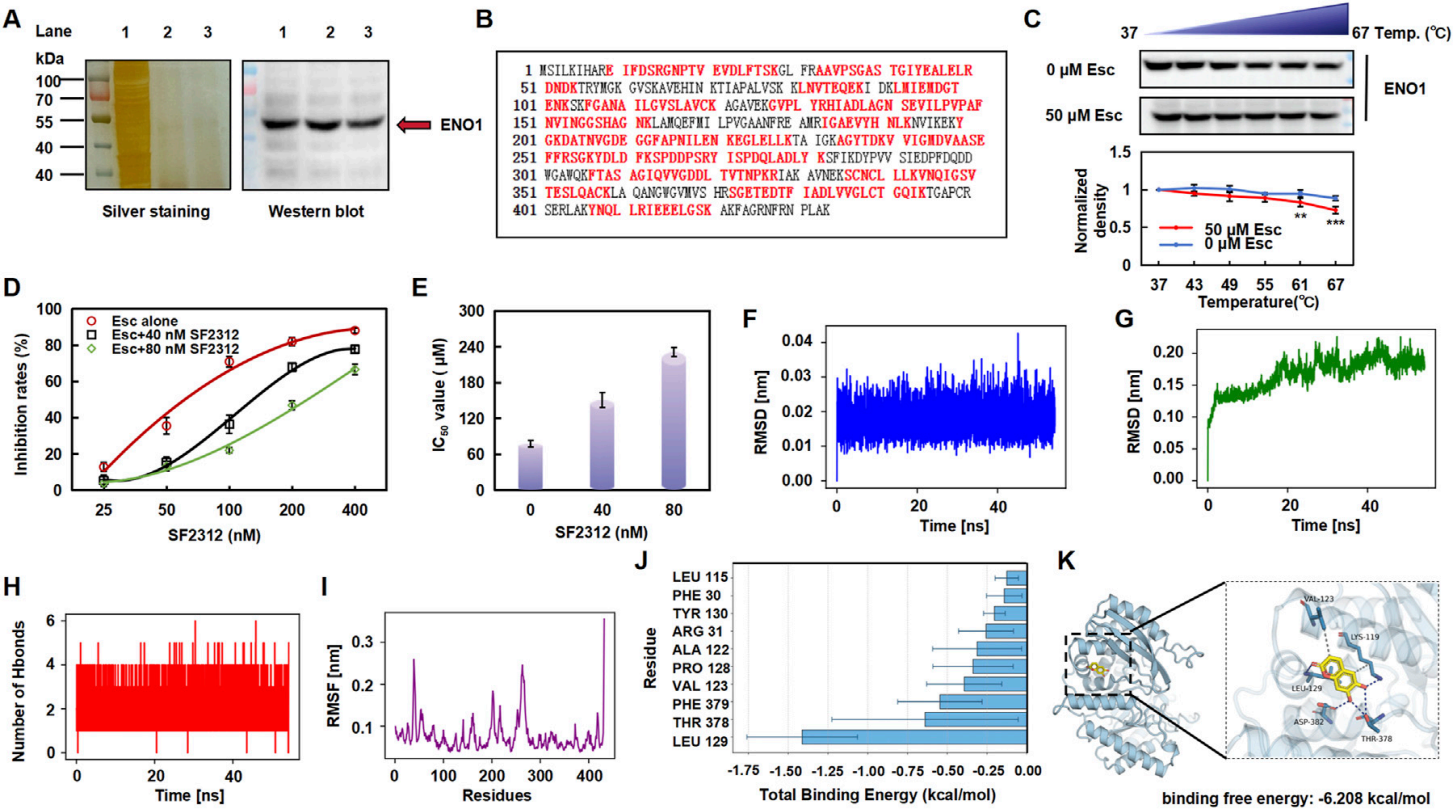
Esc targeted ENO1 and altered its stability. **(A)** Silver staining and Western blotting analysis (Lane 1: HCT116 lysate; Lane 2: 100 μM Esc-treated group; Lane 3: 200 μM Esc-treated group). **(B)** Sequence of the ENO1 protein and the detected peptide (highlighted in red). **(C)** Treatment with 50 µM Esc reduced the thermal stability of ENO1, as assessed by CETSA (n = 3). **(D,E)** Cells were pre-treated with SF2312 (40 nM or 80 nM) for 4 h, and then they were treated with Esc for 72 h. The inhibition and IC_50_ value of Esc were assayed. **(F)** RMSD of the Esc. **(G)** RMSD of the complex. **(H)** Number of hydrogen bonds between Esc and ENO1. **(I)** RMSF fluctuation diagram of the ENO1. **(J)** Analysis of the binding free energy contributions of the top ten amino acids. **(K)** Molecular docking analysis (ENO1 in blue, Esc in yellow). Data are expressed as means ± SD. *p < 0.05, **p < 0.01 versus the Ctrl group.

A DARTS assay was conducted to identify potential targets of Esc, revealing a protein band with a molecular weight ranging between 40 kDa and 50 kDa (Figure 5A, left). Thus, the isolated protein was subjected to enzymatic hydrolysis and was identified as ENO1 through HPLC-MS/MS, achieving a 64% coverage rate (see Figure 5B). Western blot analysis corroborated the presence of ENO1 in the captured protein (Figure 5A, right, lane 2), while CETSA demonstrated that the interaction altered the thermal stability of ENO1 (Figure 5C). Then, the HCT116 cells were exposed to SF2312, an inhibitor of ENO1, to further verify that ENO1 is the target of Esc. The cells became dose-dependently less sensitive to the Esc concentration after the SF2312 pretreatment (Figures 4D,E), confirming that ENO1 was the key target protein.

Next, we conducted molecular dynamics simulations, employing the root-mean-square deviation (RMSD) to measure the degree of conformational differences or the stability of the trajectory. As shown in Figures 4F,G, the RMSD fluctuations of the ligand are relatively small (<0.02 nm); the overall RMSD of the ENO1 complex rises from its initial value and fluctuates approximately 0.19 nm during the simulation, indicating the stability of the complex. The analysis of hydrogen bonds shows that the number of hydrogen bonds between Esc and ENO1 remains stable throughout the simulation (Figure 5H). The root mean square fluctuation (RMSF) analysis reveals that the overall RMSF values of ENO1 protein’s amino acids are relatively low (<0.3 nm), indicating a more rigid binding site that helps maintain the stability of ligand binding (Figure 5I).

Meanwhile, the molecular docking analysis showed that Esc formed hydrogen bonds with the LEU-129, LYS-119, THR-378, and ASP-382 sites of ENO1 (binding free energy: 6.208 kcal/mol). Among the top 10 amino acids involved in the binding of Esc to ENO1, LEU-129 was identified as the key residue contributing the most to ligand binding, likely forming stable interactions with Esc through hydrophobic effects (Figure 5J).

### 3.6 Esc inhibited tumor growth and downregulated ENO1 expression *in vivo*

**FIGURE 6:**
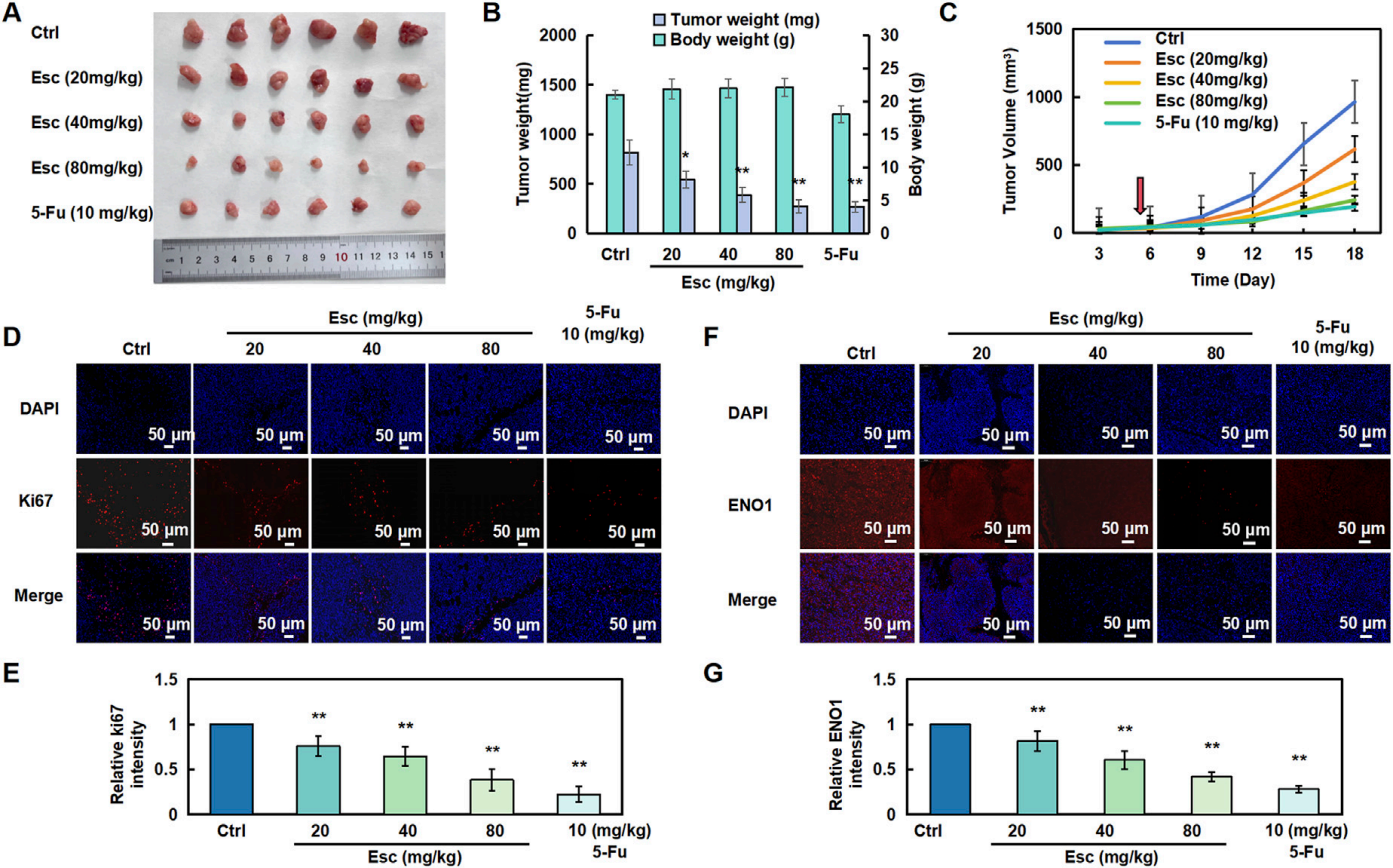
Esc inhibited the proliferation of HCT116 cells *in vivo*. **(A–C)** Nude mice were subcutaneously implanted with HCT116 cells and subsequently treated with 5-Fu (10 mg/kg/2 days, serving as a positive control) and Esc (20 mg/kg/day, 40 mg/kg/day, and 80 mg/kg/day) for 13 days after tumor formation (the red arrow at day 5 represents the first day of treatment). Tumor size was recorded (n = 6). **(D,E)** Immunofluorescent staining for Ki67 was performed to assess cell proliferation. Histograms display the relative Ki67 intensity (n = 6). **(F,G)** Immunofluorescent staining of ENO1 in tumor tissues. Histograms depict the relative ENO1 intensity (n = 6). Data are presented as means ± SD. *p < 0.05, **p < 0.01 versus the Ctrl group.

To investigate the inhibitory effect of Esc on HCT116 cells *in vivo*, a subcutaneous transplantation tumor model was established in nude mice. As shown in Figures 5A–C, the tumor growth rate was significantly lower in the Esc group in the xenograft tumor model than in the Ctrl group. In addition, immunofluorescent staining for Ki67 (red, a marker of tumor proliferation) was performed, and the results showed a significant decrease in the number of Ki67-positive cells in tumor tissues (Figures 6D,E). Moreover, ENO1 expression was examined in tumor tissues, and the results uncovered that Esc suppressed ENO1 accumulation in a concentration-dependent manner compared with the Ctrl group (Figures 6F,G). Overall, these findings suggest that Esc not only significantly inhibits HCT116 cell growth *in vivo* but also downregulates ENO1 expression.

### 3.7 Silencing ENO1 reversed the anti-CRC effects of Esc by modulating the PI3K/akt/Stat3/mTOR signaling pathway

**FIGURE 7:**
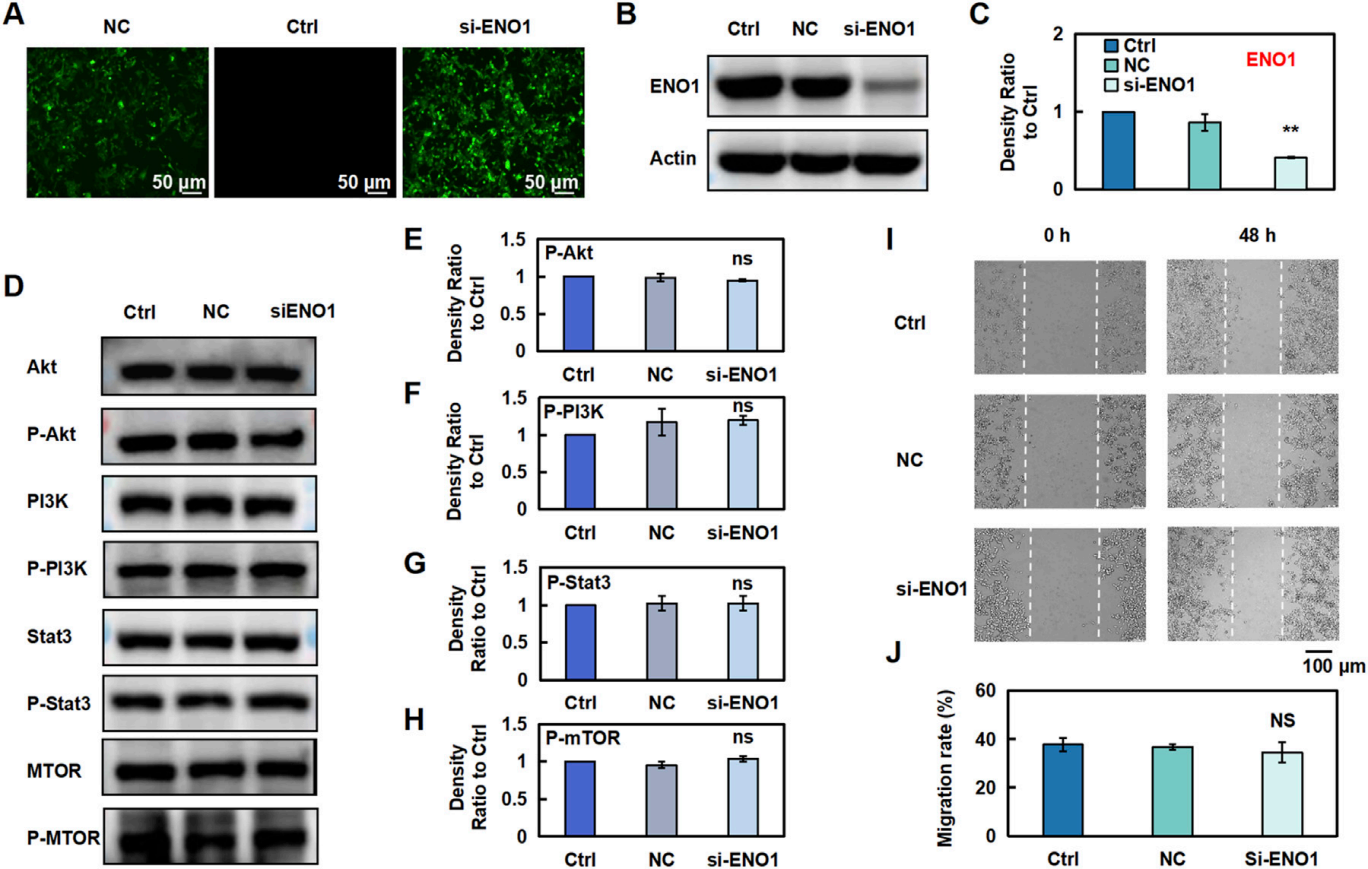
Esc exerts anti-CRC effects by inhibiting ENO1 expression. **(A–C)** HCT116 Cells were transfected with recombinant ENO1 retroviral vectors or NC virus for 72 h. The effect of recombinant retrovirus on ENO1 expression was assessed using fluorescence microscopy and Western blotting. Histograms show the intensity ratio (n = 3). **(D–H)** Cells were transfected with recombinant ENO1 retroviral vectors and NC virus, and the expressions of specific proteins were detected. Histograms show the density ratio (n = 3). **(I,J)** Analysis of the migration rate of HCT116 cells, as displayed on the histogram (n = 3). Data are expressed as means ± SD. *p < 0.05, **p < 0.01 versus the Ctrl group.

To evaluate the effect of ENO1 on the anti-CRC effects of Esc in HCT116 cells, ENO1 expression was knocked out using recombinant ENO1 retroviral vectors, achieving a transfection efficiency of 80% (Figures 7A–C). Indeed, ENO1 protein levels were markedly lower in the siENO1 group than in the negative control (NC) group. Next, the influence of Esc on the PI3K/Akt/Stat3/mTOR signaling pathway and ENO1 expression was examined, and the results indicated that ENO1 knockdown reversed the inhibitory effect of Esc on the protein levels of p-Akt, p-PI3K, p-Stat3, and p-mTOR (Figures 7D–H). Meanwhile, the migration rate of the si-ENO1 group was significantly increased, as evidenced by the results of the wound-healing assay (Figures 7I,J). Collectively, these findings indicate that ENO1 plays a crucial role in mediating the anti-CRC effects of Esc and suppressing the PI3K/Akt/Stat3/mTOR signaling pathway.

### 3.8 Esc alleviates cardiotoxicity induced by 5-FU

**FIGURE 8:**
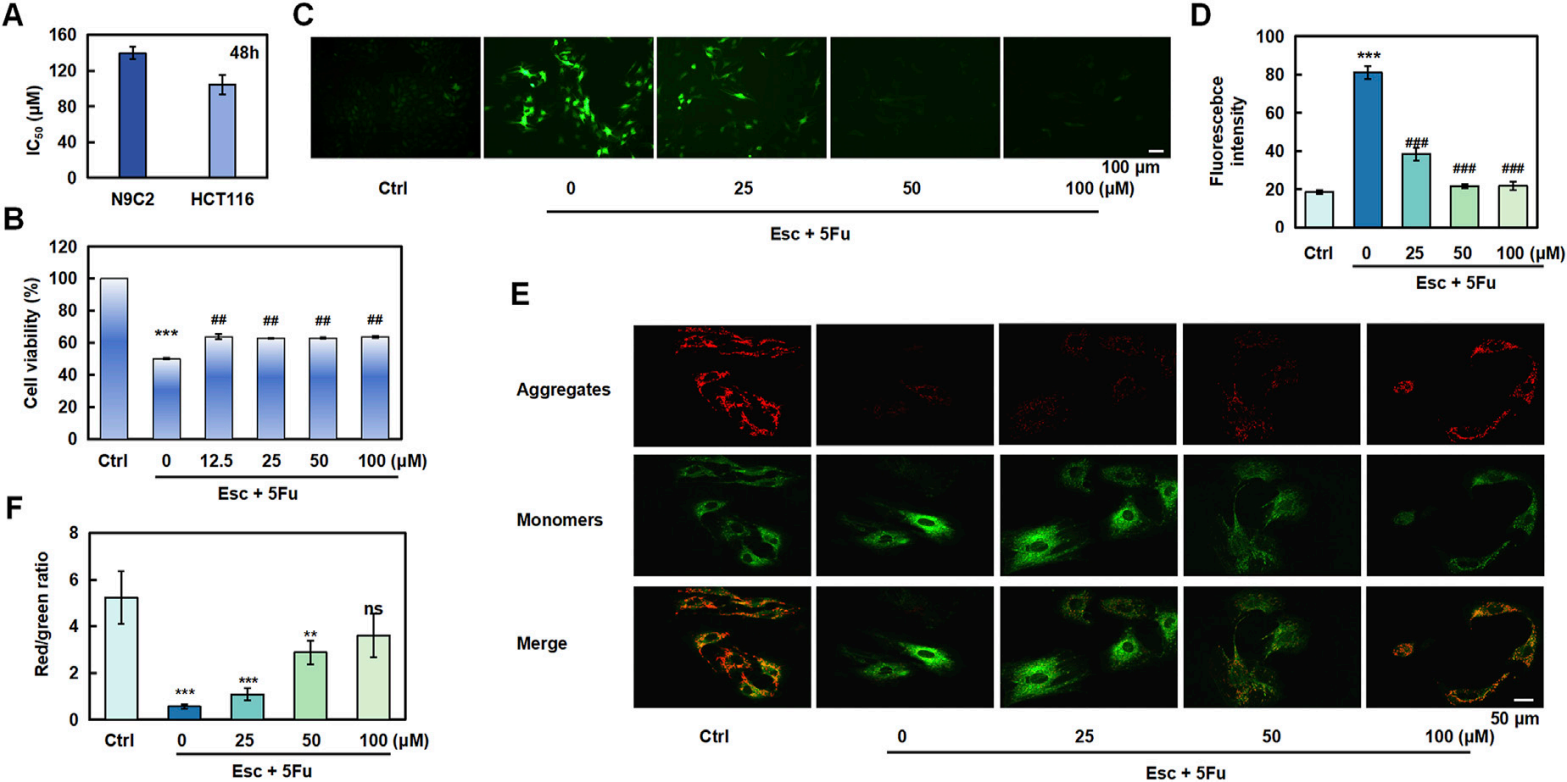
The protective effect of esculetin against 5-FU-induced cardiotoxicity. **(A)** H9C2 and HCT 116 cells were exposed to Esc (0–400 μM) for 48 h, and the IC_50_ values were determined. **(B)** The effect of Esc on the viability of H9C2 cells stimulated by 5-Fu. **(C,D)** The effect of Esc on intracellular reactive ROS in H9C2 cells stimulated by 5-Fu. **(E,F)** The effect of Esc on mitochondrial membrane potential in H9C2 cells following stimulation by 5-Fu. Data are expressed as means ± SD. ***p < 0.001 versus the Ctrl group, ^##^ *p* < 0.01, ^###^ *p* < 0.001 versus the 5Fu group.

To investigate Esc’s potential in alleviating chemotherapy drug-induced cardiomyocyte injury, we initially evaluated its effects on H9C2 cell proliferation. The results revealed that Esc exhibited relatively low cardiotoxicity, with an IC_50_ value of 139.68 μM against H9C2 cells following 48 h exposure (Figure 8A). We then investigated the effect of Esc on the proliferation of H9C2 cells induced by 5-Fu. Compared with the Ctrl group, the cell proliferation ability of the 5-Fu group was significantly reduced, and the Esc group decreased this phenomenon (Figure 8B). Next, we used the DCFH-DA fluorescent probe to assess reactive oxygen species (ROS) levels. We observed the intensified green fluorescence in the 5-Fu group relative to the Ctrl group, indicative of elevated ROS production. Conversely, the Esc group effectively suppressed 5-Fu-induced ROS accumulation, suggesting its protective role against oxidative damage (Figures 8C,D). Furthermore, mitochondrial functional analysis revealed that 5-Fu exposure decreased membrane potential and fluorescence intensity compared with the Ctrl group, indicating mitochondrial dysfunction. Importantly, Esc reversed these effects (Figures 8E,F).

## 4 Discussion

Neoadjuvant therapy modalities for patients with advanced rectal cancer are gradually diversifying, necessitating individualized treatment strategies. The emergence of new targets and drugs has further improved therapeutic efficacy and long-term survival outcomes. Nevertheless, the development of drug resistance can substantially reduce the effectiveness of treatments in the clinical setting. Therefore, identifying new treatments and approaches is vital for enhancing patient recovery. The chemotherapy drugs employed in cancer treatment demonstrate toxic effects that may impair cardiac function and potentially induce cardiomyopathy. Notably, combination therapy regimens frequently lead to cumulative exacerbation of adverse effects (Ma et al., 2020). Emerging studies have documented that bioactive compounds derived from natural products could serve as adjuvant therapies to alleviate drug-induced cardiotoxicity (Wang et al., 2022). Consequently, systematic investigation into active components within natural products may strengthen the evidentiary foundation for developing novel antitumor agents with enhanced efficacy and reduced toxicity, which currently represents a key focus in oncotherapeutic research. Our study demonstrated that Esc significantly attenuates 5-Fu-induced cardiotoxicity by suppressing ROS generation and stabilizing mitochondrial function.

Target identification is central for elucidating the mechanisms and the pharmacological effects of drugs. For example, the DARTS assay can identify the targets of active small molecules, thereby compensating for the limitations of traditional target fishing techniques. Consequently, it has gradually emerged as the preferred method for target screening in natural products (Ren et al., 2021). Noteworthily, it offers numerous advantages, such as the ability to preserve the original characteristics of the target protein and the use of native small molecules to mitigate the risk of structural modifications that could potentially result in false-positive results (Rodriguez-Furlan et al., 2017).

ENO1, an enzyme that plays a vital role in glycolysis, is ubiquitous in most human tissues and highly expressed in numerous cancers. This versatile protein possesses oncogenic potential, enhancing the proliferative, migratory, and invasive abilities of tumor cells, thereby facilitating the progression of several cancers (Pancholi, 2001). In addition, it acts as an oncoprotein that disrupts glucose metabolism, which in turn promotes tumor proliferation and dissemination. Its presence on the cell membrane positions it as an excellent marker for cancer prognosis and diagnosis (Huang et al., 2022). It is worthwhile emphasizing that the consistent link between elevated ENO1 levels in various cancers and poor clinical outcomes highlights its essential role in triggering cancer-promoting pathways, making it an ideal candidate for therapeutic intervention (Cancemi et al., 2019). In our study, network pharmacological analysis, transcriptomics, and DARTS techniques showed that Esc likely exerts its effects by binding with ENO1 and modulating PI3K/AKT/Stat3/mTOR signaling pathways and target processes to intervene in the development of CRC. More importantly, ENO1 reversed the anti-CRC effects of Esc through the PI3K/Akt/Stat3/mTOR signaling pathway. The above research results can provide a theoretical basis for developing new antitumor candidate drugs from the active components of natural products.

## 5 Conclusion

In summary, these results demonstrated that Esc induced cell cycle arrest and cell apoptosis, potentially by regulating the PI3K/AKT/Stat3/mTOR pathway and binding with ENO1. Additionally, Esc inhibited tumor growth by down-regulating ENO1 expression *in vivo*. Moreover, Esc alleviates cardiotoxicity induced by 5-FU. These findings provide valuable insights and highlight its potential for the treatment of CRC.

## Data Availability

The original contributions presented in the study are publicly available. This data can be found here: https://doi.org/10.5061/dryad.d7wm37qd2.
