# Supplementary material for: Esculetin inhibits the PI3K/Akt/mTOR pathway and enhances anti-colorectal cancer activity via binding to ENO1
Source: Front Pharmacol. 2025 Jul 16;16:1627114. doi: 10.3389/fphar.2025.1627114 (PMC12307286; doi:10.3389/fphar.2025.1627114)

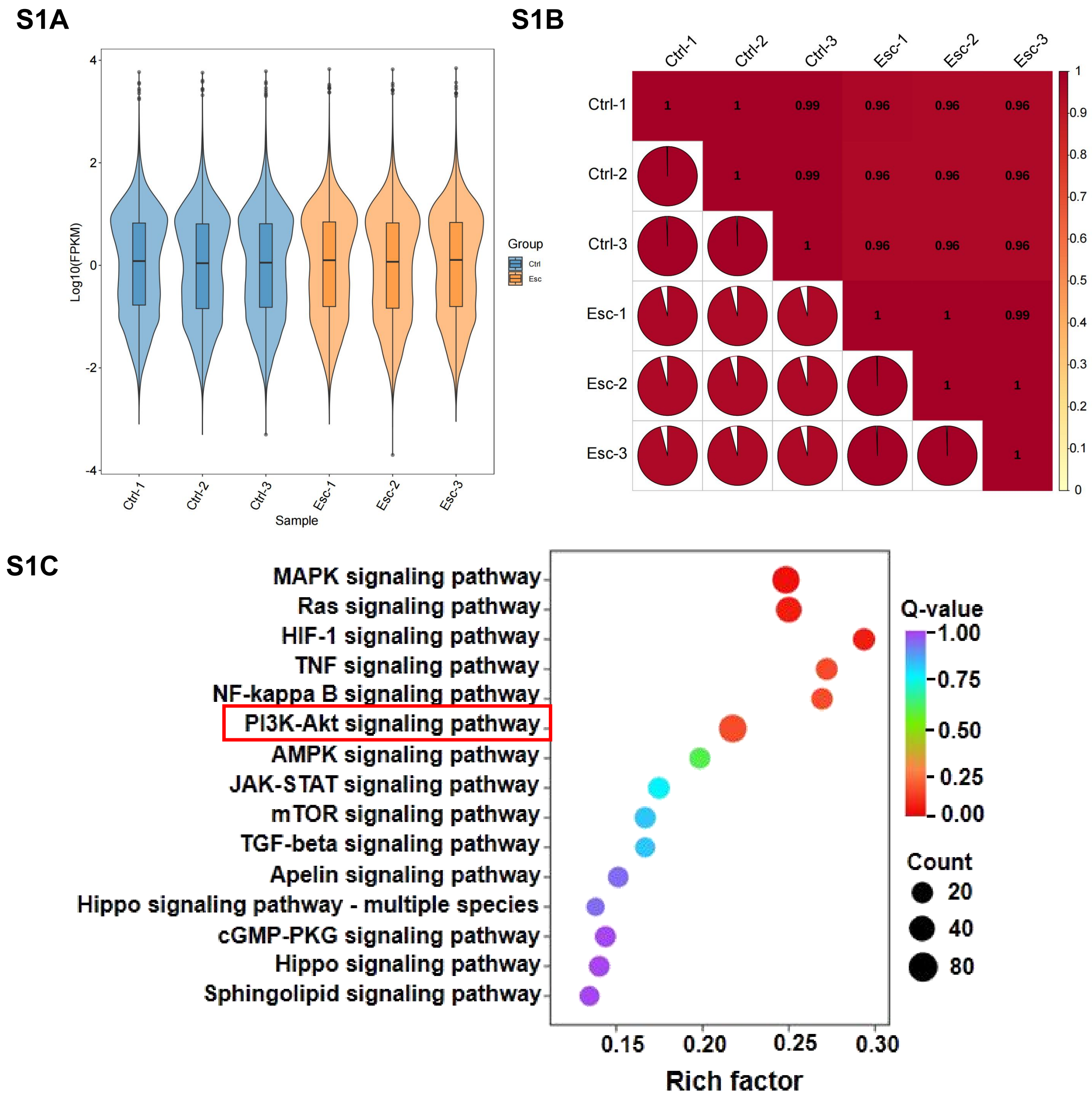
S1A
Log10(FPKM)
Sample
Ctrl-1
Ctrl-2
Ctrl-3
Esc-1
Esc-2
Esc-3
Group
Ctrl
Esc
S1B
Ctrl-1
Ctrl-2
Ctrl-3
Esc-1
Esc-2
Esc-3
S1C
MAPK signaling pathway
Ras signaling pathway
HIF-1 signaling pathway
TNF signaling pathway
NF-kappa B signaling pathway
PI3K-Akt signaling pathway
AMPK signaling pathway
JAK-STAT signaling pathway
mTOR signaling pathway
TGF-beta signaling pathway
Apelin signaling pathway
Hippo signaling pathway - multiple species
cGMP-PKG signaling pathway
Hippo signaling pathway
Sphingolipid signaling pathway
Rich factor
Q-value
Count

HCT116 细胞

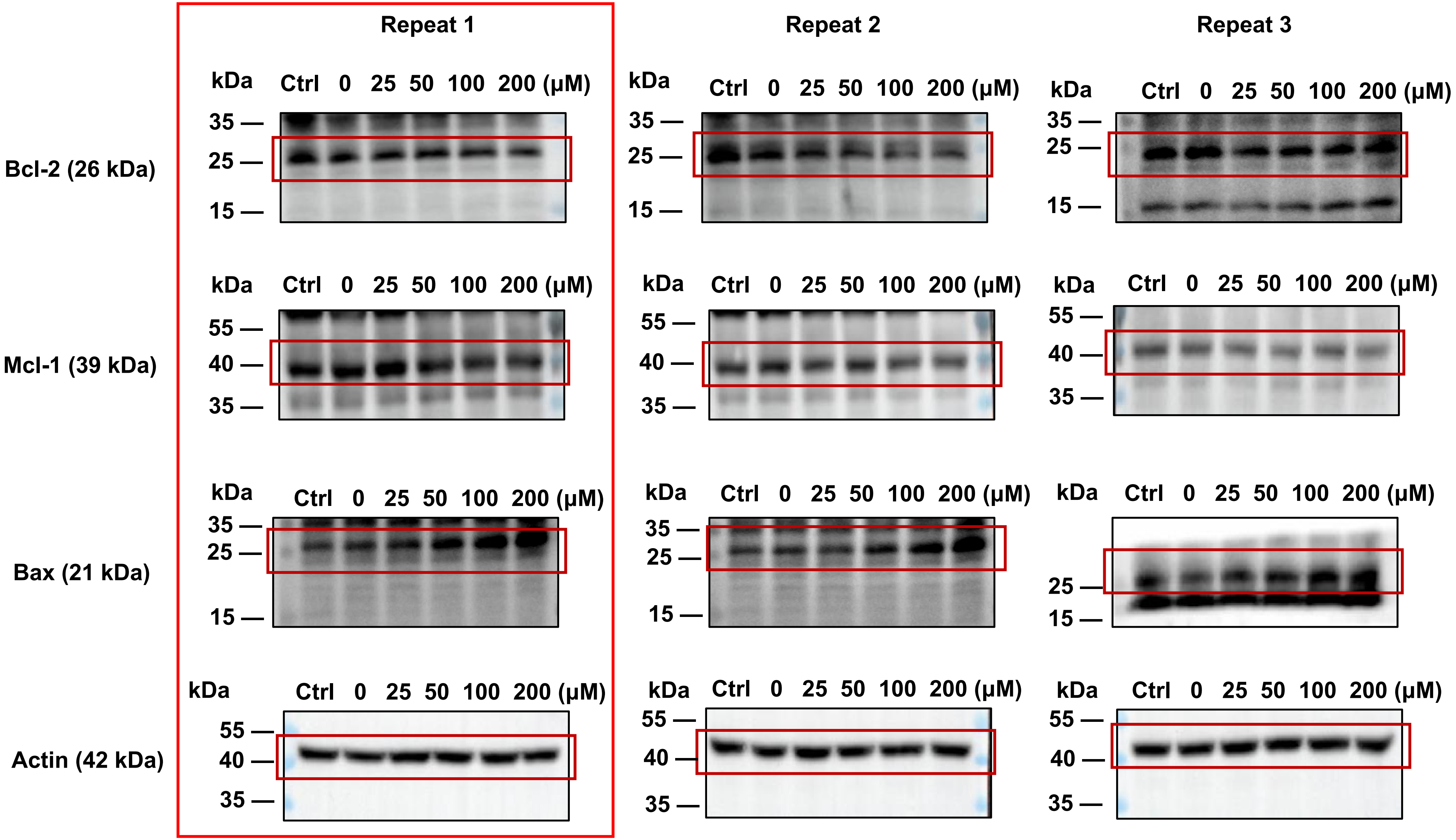
Repeat 1
Repeat 2
Repeat 3
kDa
Ctrl 0 25 50 100 200 (μM)
Bcl-2 (26 kDa)
Mcl-1 (39 kDa)
Bax (21 kDa)
Actin (42 kDa)
35
25
15
55
40

		Repeat 1			Repeat 2			Repeat 3				
Bax	Esc(μM)	intden	Normalization to Actin (with same group)	Quadratic normalization to 0 (in each group)	intden	Normalization to Actin (with same group)	Quadratic normalization to 0 (in each group)	intden	Normalization to Actin (with same group)	Quadratic normalization to 0 (in each group)	The mean value of the three normalized groups	The standard deviation of the three normalized groups
	C	14916.347	0.552631928	1	14942.86	0.634275258	1	18870.146	0.717448007	1	1	0
	D	16631.882	0.645883556	1.168740935	15898.25	0.701012298	1.105217789	18436.924	0.658555086	0.917913326	1.06395735	0.130404909
	25	17968.64	0.604337074	1.093561634	18711.59	0.704728981	1.111077521	21161.388	0.760345837	1.05979225	1.088143801	0.026068361
	50	18192.054	0.631610644	1.142913777	19676.13	0.826813436	1.303556186	22180.61	0.802713038	1.118844892	1.188438285	0.100418754
	100	21160.296	0.752796131	1.362201662	23115.78	0.861933866	1.358927145	23627.196	0.845985389	1.17915916	1.300095989	0.104747162
	200	25662.347	1.051110122	1.902007591	26414.42	0.998157553	1.573697759	26456.631	0.989595913	1.379327706	1.618344352	0.264184698
Bcl-2												
	C	19398.673	0.718696478	1	22317.85	0.947319391	1	24715.539	0.939691415	1	1	0
	D	18667.137	0.724920778	1.008660539	22264.89	0.981741289	1.036336106	26562.702	0.948802659	1.009695996	1.01823088	0.015688131
	25	17758.439	0.597267409	0.831042627	18220.76	0.686243073	0.72440518	19815.924	0.712002224	0.757697913	0.771048573	0.054557918
	50	17348.711	0.602330585	0.83808757	16384.75	0.688506152	0.726794108	18876.196	0.683126777	0.726969265	0.763950314	0.064204806
	100	17109.104	0.608671414	0.84691025	16074.27	0.599372113	0.632703309	18214.731	0.652188956	0.694045881	0.724553147	0.110313976
	200	15276.761	0.625724456	0.870637988	15468.92	0.584545278	0.617051951	17033.421	0.637125861	0.67801605	0.721901996	0.132366732
Mcl-1												
	C	25996.368	0.863612111	1	25249.49	0.86846162	1	22225.974	0.856845955	1	1	0
	D	24240.782	0.770026809	0.891635028	24933.2	0.820444771	0.944710454	23777.539	0.89498392	1.04450971	0.960285064	0.077618255
	25	21011.418	0.687065994	0.795572439	23208.61	0.841795713	0.969295238	21974.602	0.881689244	1.02899388	0.931287186	0.121263561
	50	20329.874	0.700232235	0.810817989	19960.59	0.688063109	0.792278085	20486.175	0.73525287	0.858092246	0.820396107	0.033936429
	100	18916.338	0.686846482	0.795318261	19861.83	0.684678178	0.788380467	19789.731	0.746570501	0.871300724	0.818333151	0.04600224
	200	17699.146	0.633734786	0.73381878	18702.05	0.635787942	0.73208525	19103.418	0.703271135	0.820767293	0.762223774	0.050707583
Actin												
	C	26991.468	1		23558.95	1		26301.761	1			
	D	25750.589	1		22678.98	1		27996.024	1			
	25	29732.811	1		26551.47	1		27831.267	1			
	50	28802.64	1		23797.54	1		27632.054	1			
	100	28108.933	1		26818.51	1		27928.61	1			
	200	24414.518	1		26463.18	1		26734.782	1			

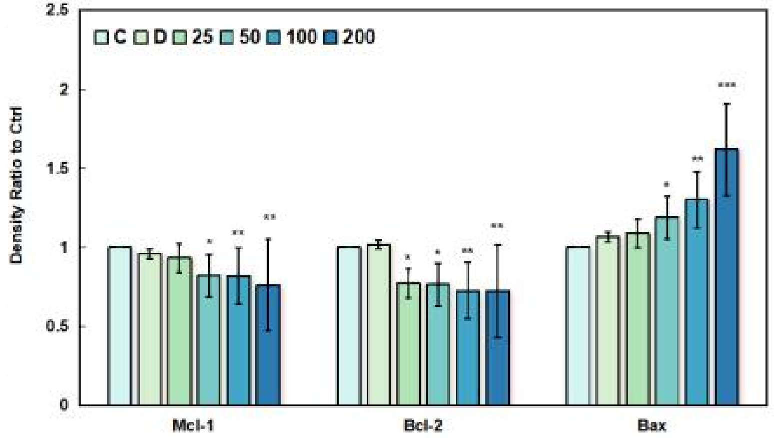

HT-29 细胞

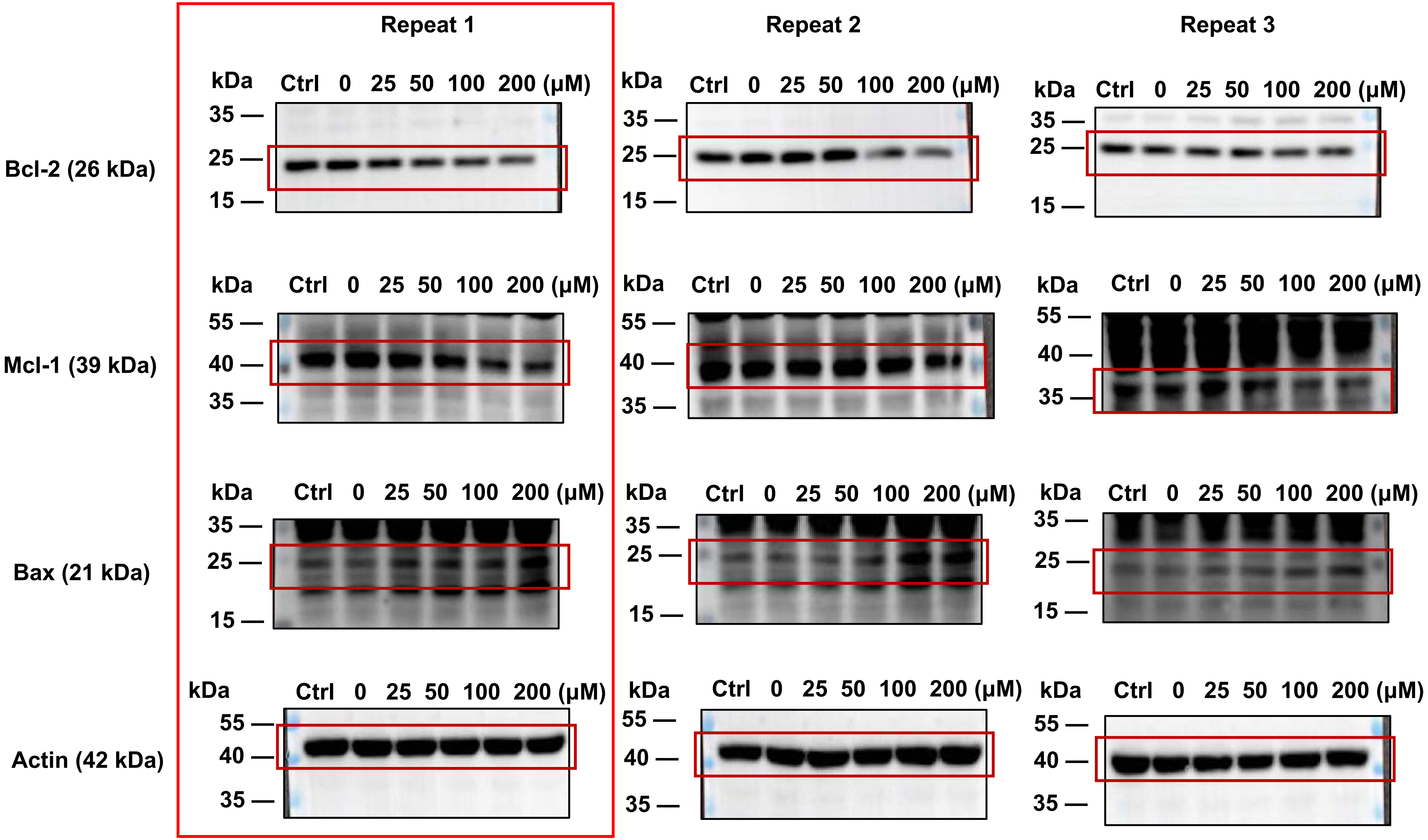

Repeat 1
Repeat 2
Repeat 3
Bcl-2 (26 kDa)
Mcl-1 (39 kDa)
Bax (21 kDa)
Actin (42 kDa)
kDa
Ctrl 0 25 50 100 200 (μM)
35
25
15
55
40

		Repeat 1			Repeat 2			Repeat 3				
Bax	Esc (μM)	intden	Normalization to Actin (with same group)	Quadratic normalization to 0 (in each group)	intden	Normalization to Actin (with same group)	Quadratic normalization to 0 (in each group)	intden	Normalization to Actin (with same group)	Quadratic normalization to 0 (in each group)	The mean value of the three normalized groups	The standard deviation of the three normalized groups
	Ctrl	16041.64	0.537838033	1	16545.69	0.580839703	1	16781.347	0.592729481	1	1	0
	0	16564.953	0.560647225	1.042409036	15247.347	0.589749614	1.015339707	15571.054	0.555761363	0.937630708	0.998459817	0.054390463
	25	17890.125	0.680609502	1.265454394	18553.589	0.655342733	1.128267799	18812.761	0.695041516	1.172611685	1.18877796	0.070007512
	50	18899.953	0.721183395	1.340893264	18293.368	0.665179083	1.145202505	18337.832	0.653114424	1.101876058	1.195990609	0.127345514
	100	21277.296	1.165428771	2.166876828	20519.903	0.851498817	1.465979018	21025.589	0.846450629	1.428055556	1.686970467	0.416043427
	200	26624.439	1.271550076	2.364187725	24272.589	1.006701712	1.733183367	25839.882	1.007631636	1.699985689	1.93245226	0.374262148
Bcl-2												
	Ctrl	24005.933	0.847907156	1	23463.66	0.860132427	1	27042.054	0.995076255	1	1	0
	0	23813.589	0.849953554	1.002413469	23439.004	0.933919071	1.085785213	25997.397	0.946556855	0.95124052	1.013146401	0.067911453
	25	22013.539	0.81329495	0.95917925	22394.175	0.855842103	0.995012019	22879.711	0.797085133	0.801029196	0.918406822	0.103218831
	50	21432.832	0.763344965	0.900269516	20583.589	0.767127448	0.891871325	19692.933	0.704356956	0.70784219	0.833327677	0.108754715
	100	19692.146	0.682813569	0.805292848	17932.974	0.619446789	0.720176067	16913.539	0.56768074	0.570489685	0.698652867	0.118872063
	200	18000.782	0.651160036	0.767961482	16534.296	0.585422684	0.680619246	15791.861	0.524538936	0.527134411	0.658571713	0.121917963
Mcl-1												
	Ctrl	26374.69	0.792762979	1	22113.66	0.810644036	1	23371.882	0.860023607	1	1	0
	0	28161.447	0.921624118	1.162546867	22587.146	0.899977081	1.110200089	21677.983	0.789288382	0.917751996	1.063499651	0.128906285
	25	23998.368	0.80428132	1.014529362	21698.832	0.829268058	1.022974352	22118.811	0.77057684	0.895994987	0.9778329	0.070999384
	50	21071.175	0.757780201	0.955872336	20809.953	0.802264024	0.989662525	21340.861	0.763298382	0.887531895	0.944355586	0.052030213
	100	18376.175	0.657653471	0.829571371	17197.347	0.662711301	0.817512091	19861.205	0.666615281	0.77511277	0.807398744	0.028603228
	200	17250.811	0.629901582	0.794564829	16542.418	0.630347112	0.777588046	16474.589	0.547216277	0.636280531	0.736144469	0.086900273
Actin												
	Ctrl	29826.154	1		27279.125	1		27175.861	1			
	0	29546.125	1		25097.468	1		27465.225	1			
	25	26285.447	1		26166.246	1		28704.225	1			
	50	26206.861	1		25939.033	1		27958.74	1			
	100	18257.054	1		25949.983	1		29794.104	1			
	200	20938.569	1		26243.347	1		30106.175	1			

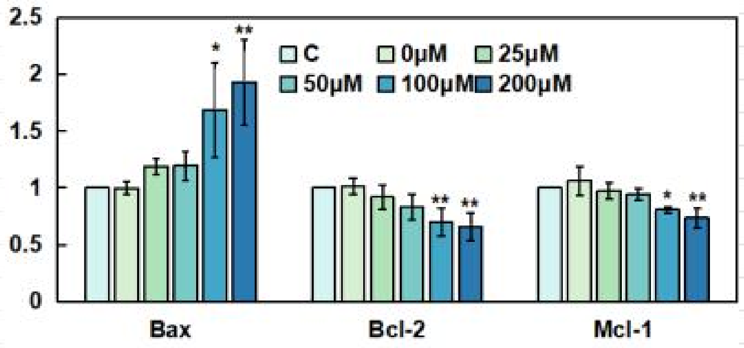

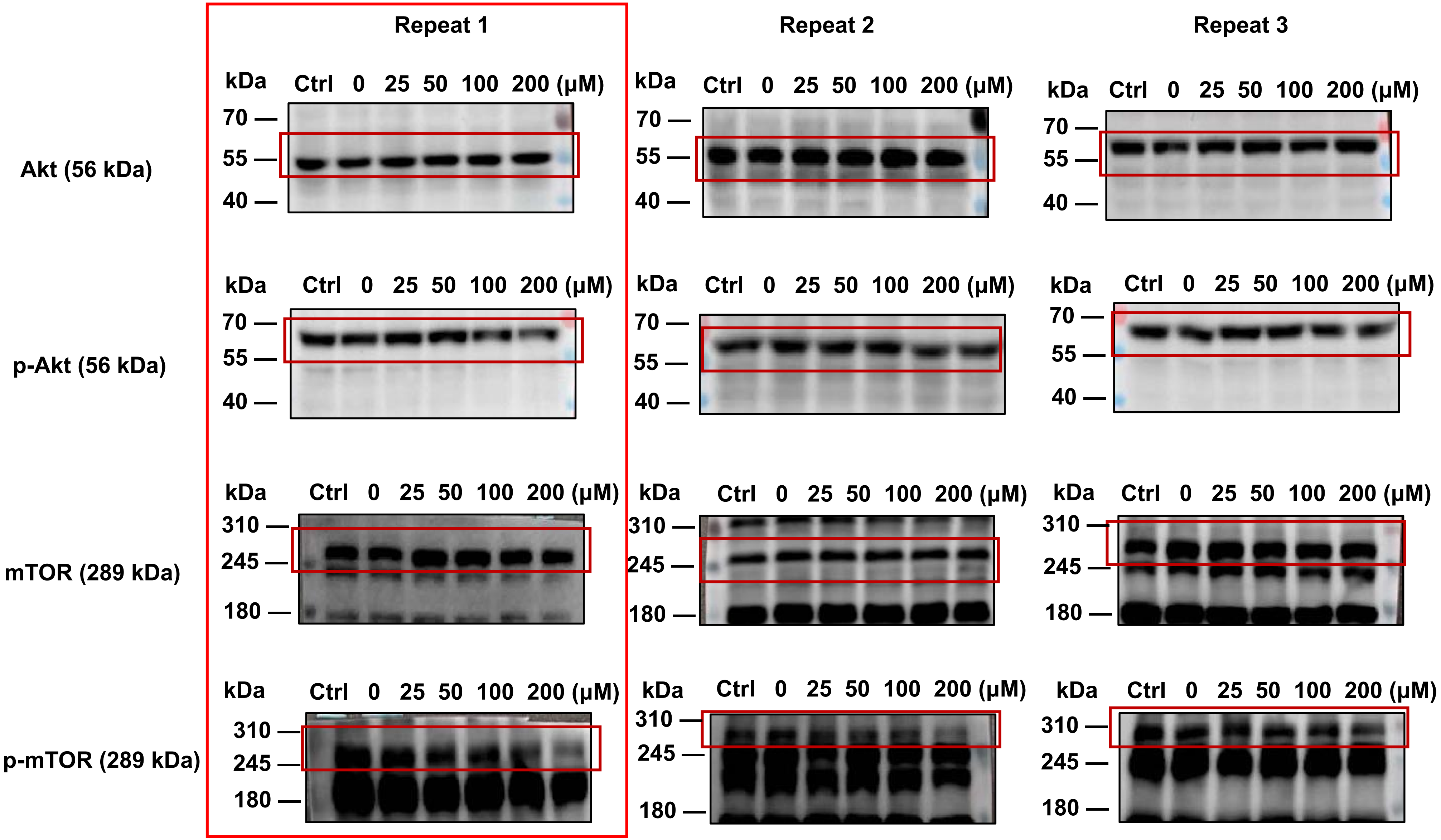

Repeat 1
Repeat 2
Repeat 3
Akt (56 kDa)
p-Akt (56 kDa)
mTOR (289 kDa)
p-mTOR (289 kDa)
kDa
Ctrl 0 25 50 100 200 (μM)
70
55
40
310
245
180

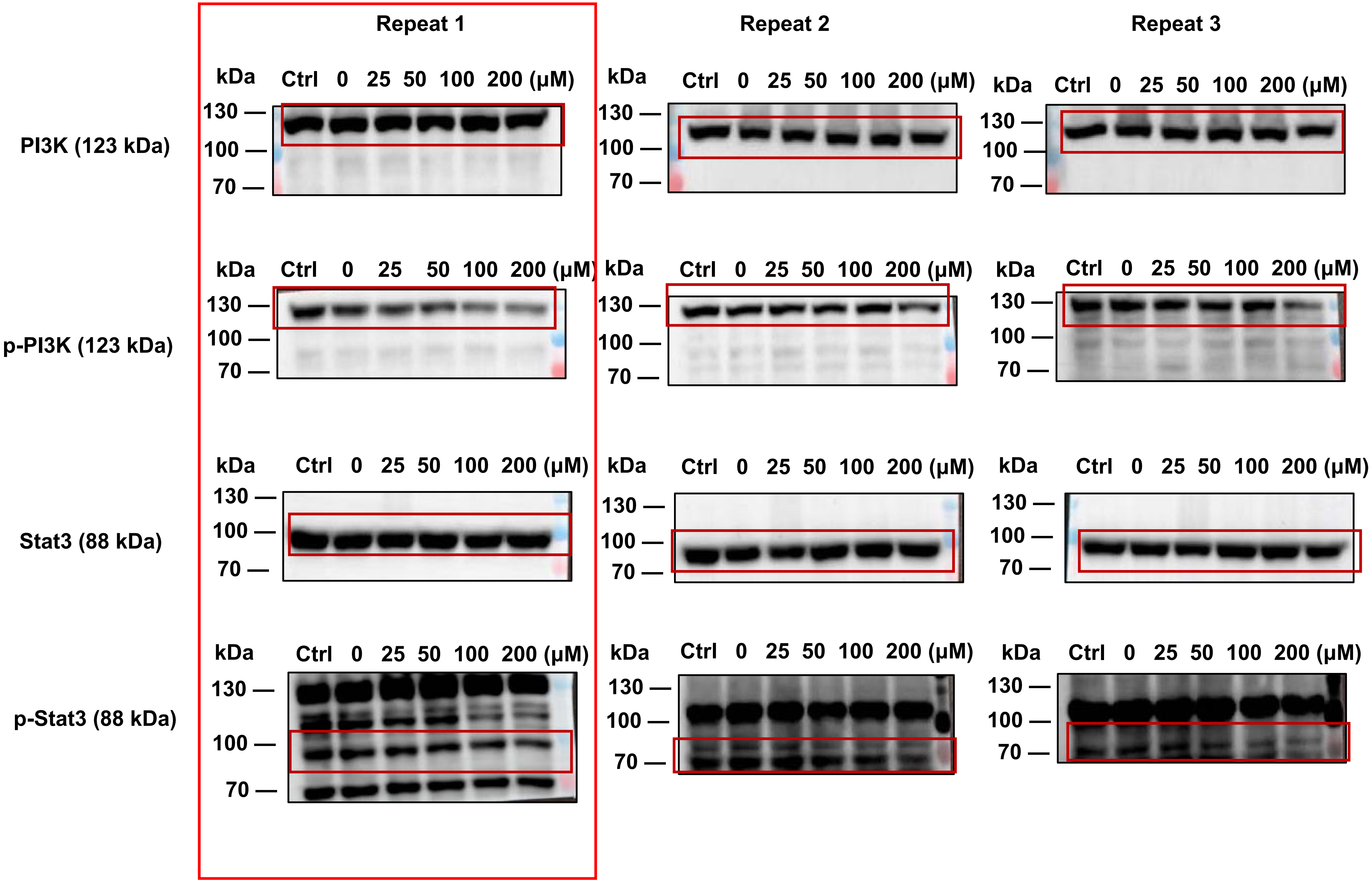

Repeat 1
Repeat 2
Repeat 3
PI3K (123 kDa)
p-PI3K (123 kDa)
Stat3 (88 kDa)
p-Stat3 (88 kDa)
kDa
Ctrl 0 25 50 100 200 (μM)
130
100
70

P-Akt		Repeat 1			Repeat 2			Repeat 3				
		intden	Normalization to Actin (with same group)	Quadratic normalization to 0 (in each group)	intden	Normalization to Actin (with same group)	Quadratic normalization to 0 (in each group)	intden	Normalization to Actin (with same group)	Quadratic normalization to 0 (in each group)	The mean value of the three normalized groups	The standard deviation of the three normalized groups
	Ctrl	23944.468	0.975891938	1	26622.296	1.067652919	1	25925.953	1.04114155	1	1	0
	0	25376.681	1.012515539	1.037528336	25744.075	1.053467364	0.986713328	24982.569	1.038371789	0.997339688	1.007193784	0.026802401
	25	24303.803	0.949630568	0.973089879	24510.125	0.967182379	0.905895879	24670.711	0.97817453	0.939521172	0.93950231	0.033597004
	50	23126.116	0.89793557	0.920117828	23190.054	0.917114697	0.859000786	23705.368	0.918644971	0.882343972	0.887154196	0.030841155
	100	19616.782	0.769214137	0.78821651	20705.61	0.818799656	0.766915579	20056.853	0.793818492	0.762450113	0.7725274	0.013769394
	200	17003.803	0.685854423	0.702797509	18519.581	0.711280656	0.66620963	18009.803	0.689853387	0.662593272	0.677200137	0.022241596
Akt												
	Ctrl	24535.983	1		24935.347	1		24901.468	1			
	0	25063.004	1		24437.468	1		24059.368	1			
	25	25592.903	1		25341.782	1		25221.175	1			
	50	25754.761	1		25285.882	1		25804.711	1			
	100	25502.368	1		25287.761	1		25266.296	1			
	200	24792.146	1		26036.953	1		26106.711	1			

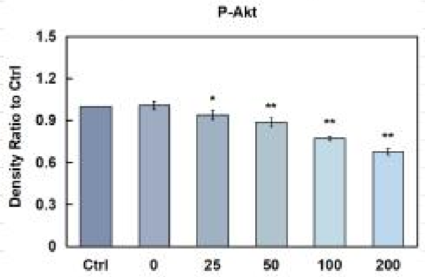

		Repeat 1			Repeat 2			Repeat 3				
P-mTOR	Esc（μM）	intden	Normalization to Actin (with same group)	Quadratic normalization to 0 (in each group)	intden	Normalization to Actin (with same group)	Quadratic normalization to 0 (in each group)	intden	Normalization to Actin (with same group)	Quadratic normalization to 0 (in each group)	The mean value of the three normalized groups	The standard deviation of the three normalized groups
	Ctrl	29129.933	1.074534661	1	23924.489	1.043855612	1	24088.589	0.982500314	1	1	0
	0	27561.246	1.097059679	1.020962579	24120.418	1.036145694	0.992613999	24949.61	0.982867195	1.000373415	1.004649998	0.014650166
	25	25669.61	0.949305222	0.883457049	22186.61	0.947838327	0.908016699	23906.953	0.947209648	0.964080759	0.918518169	0.041325008
	50	23386.53	0.870535318	0.810150988	21561.489	0.883575639	0.846453886	21386.903	0.911820227	0.928061003	0.861555293	0.06038818
	100	19727.388	0.799521148	0.744062687	20506.175	0.874864959	0.838109168	19386.903	0.813696725	0.828189786	0.803453881	0.051672855
	200	13687.589	0.576952776	0.53693268	17104.882	0.739702432	0.708625238	14842.246	0.602170008	0.612895486	0.619484468	0.086035717
mTOR												
	Ctrl	27109.347	1		22919.347	1		24517.64	1			
	0	25122.832	1		23278.983	1		25384.518	1			
	25	27040.418	1		23407.589	1		25239.347	1			
	50	26864.539	1		24402.539	1		23455.175	1			
	100	24674.004	1		23439.246	1		23825.711	1			
	200	23723.933	1		23124.004	1		24647.933	1			

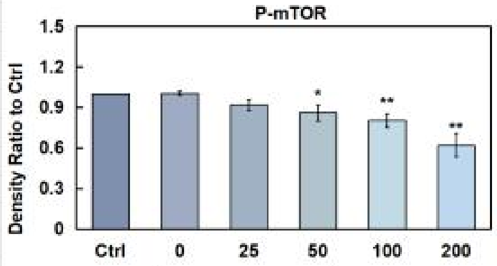

		Repeat 1			Repeat 2			Repeat 3				
P-PI3K	Esc（μM）	intden	Normalization to Actin (with same group)	Quadratic normalization to 0 (in each group)	intden	Normalization to Actin (with same group)	Quadratic normalization to 0 (in each group)	intden	Normalization to Actin (with same group)	Quadratic normalization to 0 (in each group)	The mean value of the three normalized groups	The standard deviation of the three normalized groups
	Ctrl	27828.104	0.999294126	1	26005.246	1.018253245	1	24084.761	0.995153593	1	1	0
	0	26969.983	0.966069622	0.966752027	25538.296	1.031164234	1.012679546	23246.832	0.965292166	0.969993148	0.983141573	0.025631915
	25	24633.711	0.912811339	0.913456124	21050.075	0.869962984	0.854367995	19761.64	0.829275564	0.833314143	0.867046087	0.041547982
	50	22959.782	0.857554991	0.858160744	18990.56	0.761939213	0.748280663	19408.418	0.826202151	0.830225763	0.81222239	0.057109536
	100	21680.74	0.783936475	0.784490226	15833.075	0.642705106	0.631183951	19092.874	0.780404121	0.784204696	0.733292957	0.088429109
	200	18362.095	0.665868791	0.666339142	13258.803	0.548121359	0.538295716	13891.874	0.599434829	0.602354082	0.602329647	0.064021717
PI3K												
	Ctrl	27847.761	1		25539.075	1		24202.054	1			
	0	27917.225	1		24766.468	1		24082.69	1			
	25	26986.64	1		24196.518	1		23830.004	1			
	50	26773.539	1		24923.983	1		23491.125	1			
	100	27656.246	1		24635.054	1		24465.368	1			
	200	27576.146	1		24189.539	1		23174.953	1			

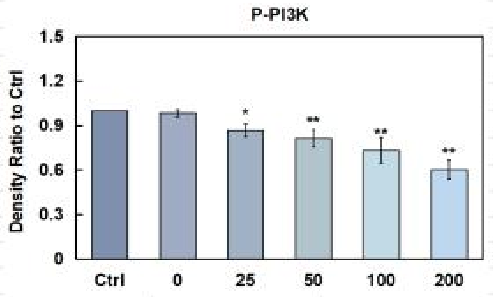

		Repeat 1			Repeat 2			Repeat 3				
p-Stat3	Esc（μM）	intden	Normalization to Actin (with same group)	Quadratic normalization to 0 (in each group)	intden	Normalization to Actin (with same group)	Quadratic normalization to 0 (in each group)	intden	Normalization to Actin (with same group)	Quadratic normalization to 0 (in each group)	The mean value of the three normalized groups	The standard deviation of the three normalized groups
	Ctrl	25410.296	0.930080333	1	26295.347	0.958960077	1	24556.196	0.895535306	1	1	0
	0	26931.69	1.02153068	1.098325213	25582.004	1.008705376	1.051874212	25192.225	0.948458702	1.059096941	1.069765455	0.024995735
	25	23848.368	0.905715066	0.973803051	22846.832	0.885643156	0.923545388	23380.61	0.872512332	0.974291383	0.957213274	0.029158267
	50	22414.368	0.791056068	0.850524455	21436.075	0.749692016	0.781776044	22749.388	0.795623011	0.888432881	0.84024446	0.054066431
	100	20119.903	0.756280997	0.813135135	19543.882	0.681079047	0.710226696	19541.953	0.681011824	0.760452234	0.761271355	0.051459109
	200	18690.154	0.707135093	0.760294642	16820.861	0.611823788	0.638007569	16870.196	0.613618246	0.685197157	0.694499789	0.061672005
Stat3												
	Ctrl	27320.539	1		27420.69	1		27420.69	1			
	0	26364.054	1		25361.225	1		26561.225	1			
	25	26330.983	1		25796.882	1		26796.882	1			
	50	28334.74	1		28593.175	1		28593.175	1			
	100	26603.74	1		28695.468	1		28695.468	1			
	200	26430.811	1		27492.983	1		27492.983	1			

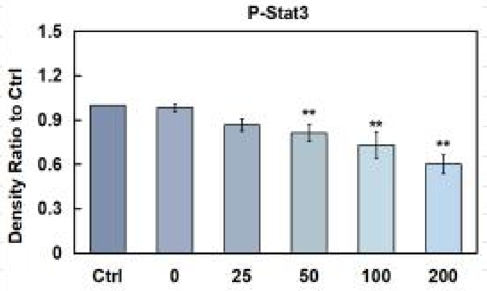

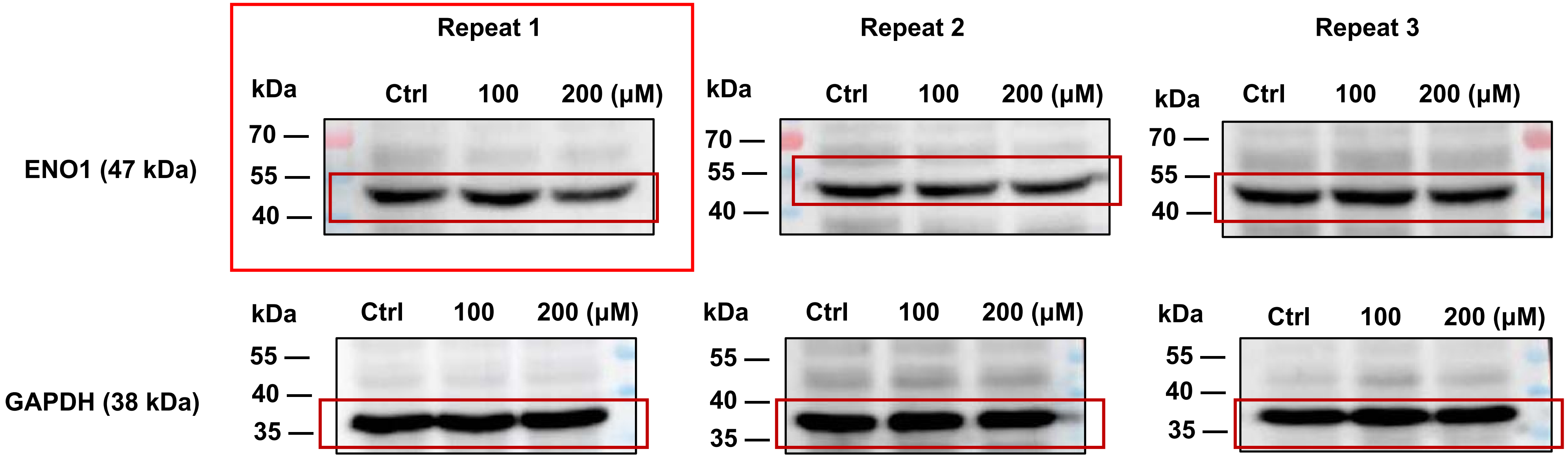

		Repeat 1			Repeat 2			Repeat 3				
ENO1	Esc (μM)	intden	Normalization to Actin (with same group)	Quadratic normalization to 0 (in each group)	intden	Normalization to Actin (with same group)	Quadratic normalization to 0 (in each group)	intden	Normalization to Actin (with same group)	Quadratic normalization to 0 (in each group)	The mean value of the three normalized groups	The standard deviation of the three normalized groups
	C	53864.794	0.989802414	1	55761.279	1.014572297	1	51971.472	1.000169737	1	1	0
	100	49053.087	0.910099093	0.919475523	44157.179	0.820303279	0.808521267	45036.865	0.873543015	0.873394767	0.867130519	0.055741747
	200	44507.179	0.806437848	0.814746293	40746.208	0.743497405	0.732818555	38106.522	0.742676432	0.742550393	0.763371747	0.044756956
GAPDH												
	C	54419.744	1		54960.38	1		51962.652	1			
	100	53898.622	1		53830.309	1		51556.551	1			
	200	55189.844	1		54803.43	1		51309.723	1			

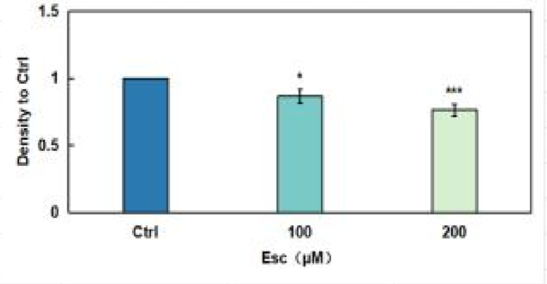

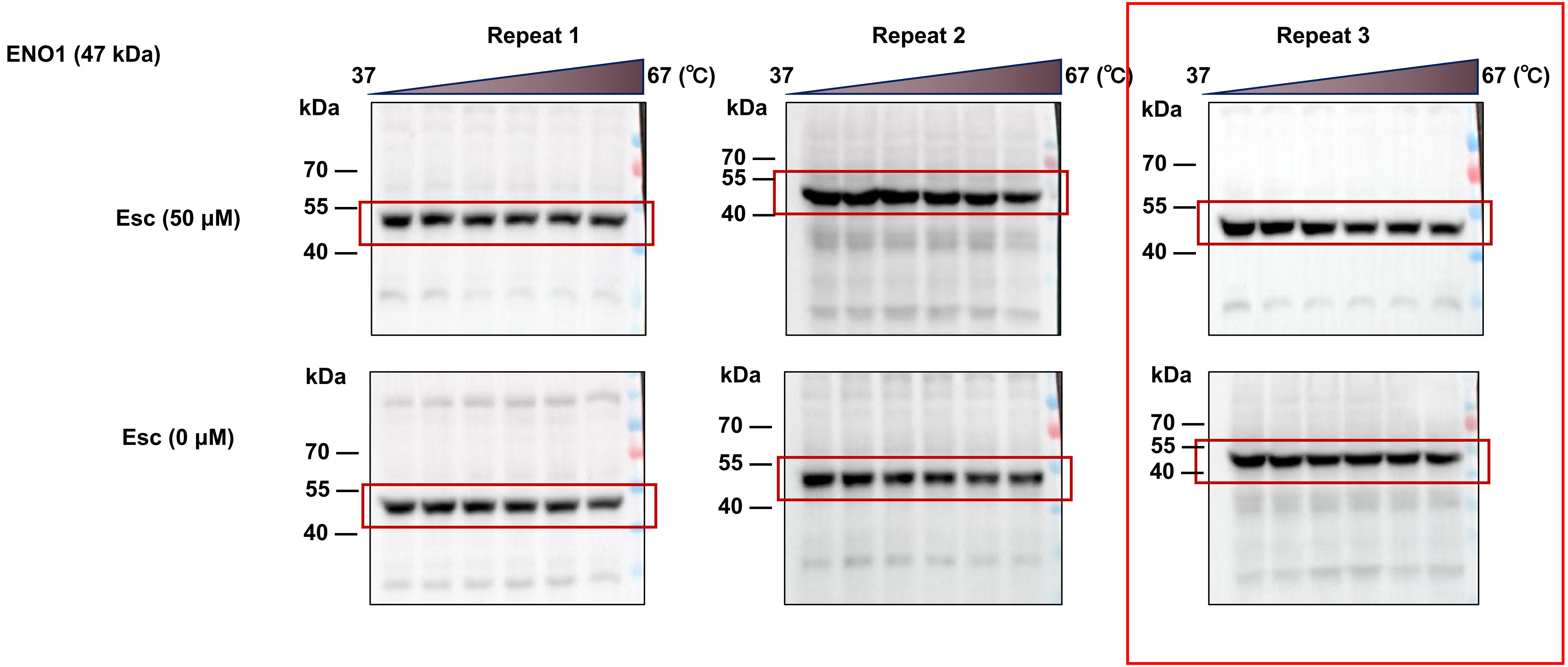

ENO1 (47 kDa)
Repeat 1
Repeat 2
Repeat 3
37
67 (℃)
kDa
70
55
40
Esc (50 μM)
Esc (0 μM)

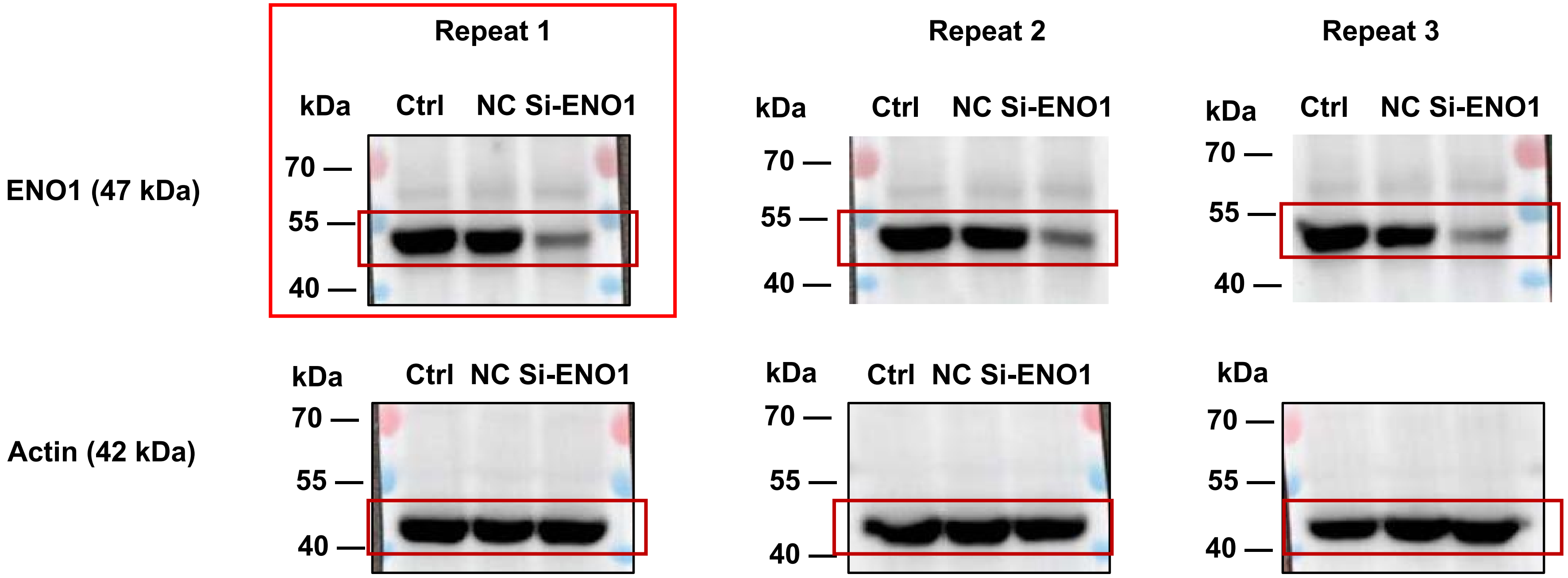

si-ENO1	Esc (μM)	Repeat 1 intden	Normalization to Actin (with same group)	Quadratic normalization to 0 (in each group)	Repeat 2 intden	Normalization to Actin (with same group)	Quadratic normalization to 0 (in each group)	Repeat 3 intden	Normalization to Actin (with same group)	Quadratic normalization to 0 (in each group)	The mean value of the three normalized groups	The standard deviation of the three normalized groups
	Ctrl	46054.66	0.994704883	1	47207.095	0.88654825	1	44973.116	0.857510211	1	1	0
	NC	41389.782	0.82888833	0.833300756	39951.66	0.872384073	0.98402323	33555.075	0.66359956	0.773867823	0.863730603	0.108331923
	si-ENO1	20417.539	0.413695	0.415897224	18501.882	0.364177801	0.410781704	16537.782	0.344180382	0.401371759	0.409350229	0.007367776
Actin												
	Ctrl	46299.823	1		53248.196	1		52446.158	1			
	NC	49934.087	1		45795.953	1		50565.246	1			
	si-ENO1	49354.087	1		50804.53	1		48049.752	1			

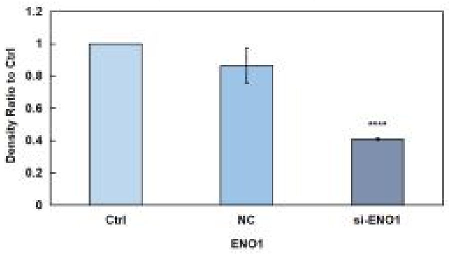

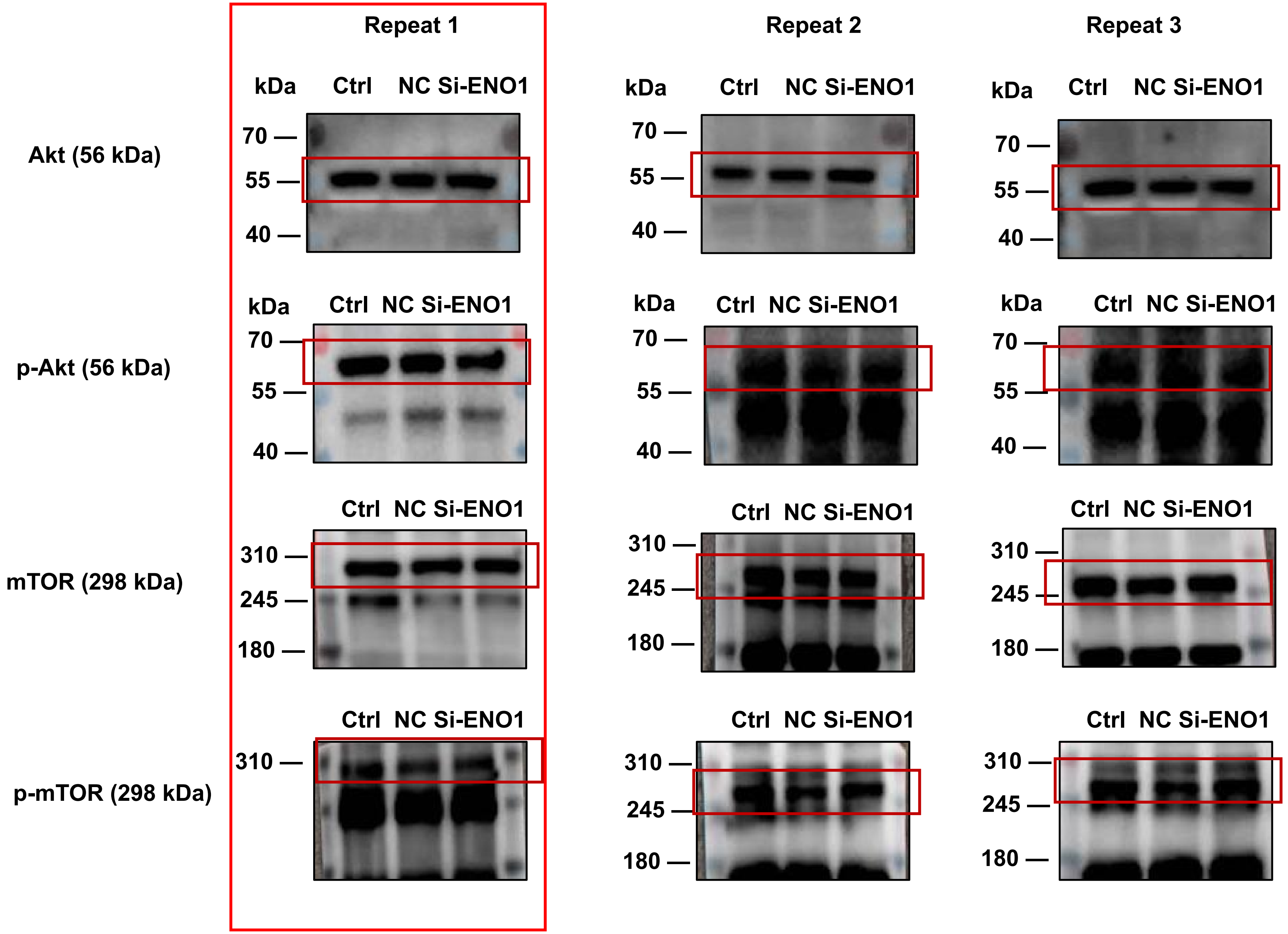

Repeat 1
Repeat 2
Repeat 3
Akt (56 kDa)
p-Akt (56 kDa)
mTOR (298 kDa)
p-mTOR (298 kDa)
kDa
Ctrl
NC
Si-ENO1
70
55
40
310
245
180

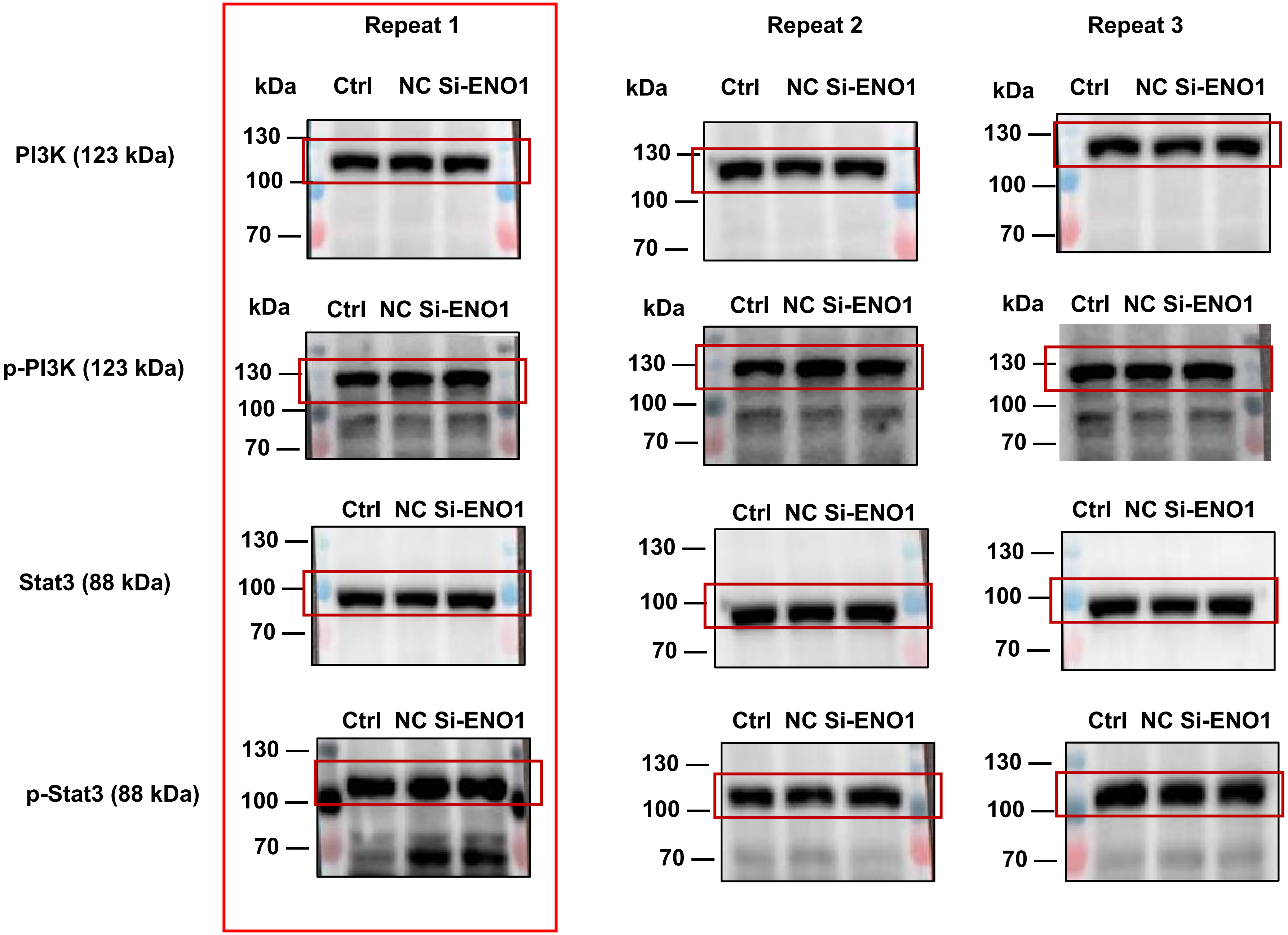
Repeat 1
Repeat 2
Repeat 3
PI3K (123 kDa)
p-PI3K (123 kDa)
Stat3 (88 kDa)
p-Stat3 (88 kDa)
kDa
Ctrl
NC
Si-ENO1
130
100
70

		Repeat 1			Repeat 2			Repeat 3				
P-Akt		intden	Normalization to Actin (with same group)	Quadratic normalization to 0 (in each group)	intden	Normalization to Actin (with same group)	Quadratic normalization to 0 (in each group)	intden	Normalization to Actin (with same group)	Quadratic normalization to 0 (in each group)	The mean value of the three normalized groups	The standard deviation of the three normalized groups
	C	42117.146	1.005441233	1	40890.338	0.967094623	1	38992.024	1.061690433	1	1	0
	NC	37900.146	0.937039704	0.931968645	36805.894	0.951162328	0.983525609	40680.874	1.100856087	1.036889899	0.984128051	0.052463221
	si-ENO1	38432.066	0.937803039	0.932727849	35993.631	0.916172815	0.947345578	37159.773	1.019639093	0.960392089	0.946821839	0.013839555
Akt												
	C	41889.217	1		42281.631	1		36726.359	1			
	NC	40446.681	1		38695.702	1		36953.853	1			
	si-ENO1	40980.957	1		39286.945	1		36444.045	1			

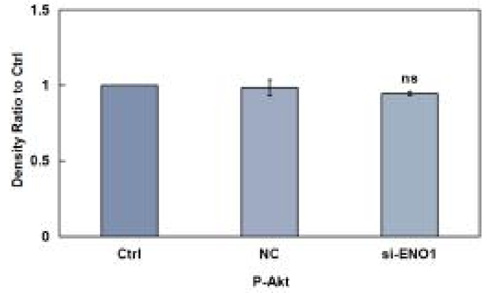

		Repeat 1			Repeat 2			Repeat 3				
P-mTOR		intden	Normalization to Actin (with same group)	Quadratic normalization to 0 (in each group)	intden	Normalization to Actin (with same group)	Quadratic normalization to 0 (in each group)	intden	Normalization to Actin (with same group)	Quadratic normalization to 0 (in each group)	The mean value of the three normalized groups	The standard deviation of the three normalized groups
	C	35459.782	0.955726983	1	33654.652	0.937793119	1	35704.711	0.931999848	1	1	0
	NC	33481.187	0.960332338	1.004818693	31205.823	0.862223497	0.919417598	32727.702	0.876748148	0.94071705	0.954984447	0.044452286
	si-ENO1	33371.539	1.028541052	1.076187103	34821.409	0.941274803	1.003712636	36513.439	0.963504001	1.033802745	1.037900828	0.036410614
mTOR												
	C	37102.418	1		35887.075	1		38309.782	1			
	NC	34864.167	1		36192.267	1		37328.51	1			
	si-ENO1	32445.51	1		36993.882	1		37896.51	1			

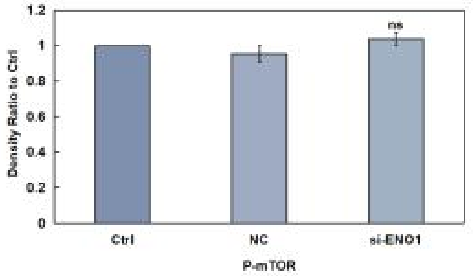

		Repeat 1			Repeat 2			Repeat 3				
P-PI3K		intden	Normalization to Actin (with same group)	Quadratic normalization to 0 (in each group)	intden	Normalization to Actin (with same group)	Quadratic normalization to 0 (in each group)	intden	Normalization to Actin (with same group)	Quadratic normalization to 0 (in each group)	The mean value of the three normalized groups	The standard deviation of the three normalized groups
	C	30438.995	0.734198756	1	36623.317	0.904316074	1	33756.803	0.81044139	1	1	0
	NC	36429.953	0.997592384	1.35874976	38192.731	1.036088008	1.145714467	32902.782	0.817924841	1.009233797	1.171232675	0.176149755
	si-ENO1	32718.61	0.920857181	1.254234188	42370.631	1.079991927	1.194263774	35000.853	0.917270074	1.131815435	1.193437799	0.061213556
PI3K												
	C	41458.794	1		40498.359	1		41652.368	1			
	NC	36517.874	1		36862.439	1		40227.146	1			
	si-ENO1	35530.602	1		39232.359	1		38157.631	1			

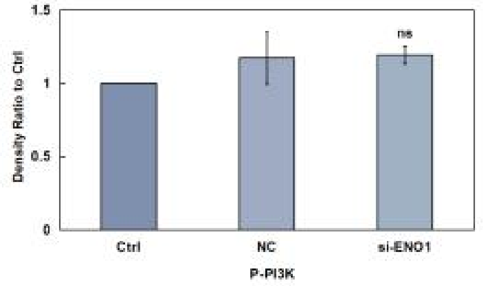

		Repeat 1			Repeat 2			Repeat 3				
P-Stat3		intden	Normalization to Actin (with same group)	Quadratic normalization to 0 (in each group)	intden	Normalization to Actin (with same group)	Quadratic normalization to 0 (in each group)	intden	Normalization to Actin (with same group)	Quadratic normalization to 0 (in each group)	The mean value of the three normalized groups	The standard deviation of the three normalized groups
	C	32236.581	0.835916877	1	34798.095	0.872373779	1	39801.267	1.038066513	1	1	0
	NC	32375.894	0.903010986	1.080264092	33201.217	0.939115472	1.076505845	35157.095	0.944603203	0.909964045	1.022244661	0.097256021
	si-ENO1	38315.903	0.886088723	1.060020138	36028.066	0.958863148	1.099142559	37701.48	0.94697909	0.912252806	1.023805168	0.098567682
Stat3												
	C	38564.338	1		39888.974	1		38341.731	1			
	NC	35853.267	1		35353.711	1		37218.903	1			
	si-ENO1	43241.61	1		37573.731	1		39812.368	1			

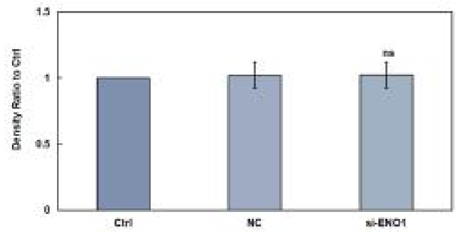

Supplement: Supplementary file 1 [file DataSheet1.pdf]
